# Complete muscle denervation triggers compensatory sprouting of a bystander tonic motor neuron in *Drosophila* larvae

**DOI:** 10.1016/j.isci.2026.116099

**Published:** 2026-07-27

**Authors:** Lizzy Olsen, Parinita Mitchelle Mandhyan, Komal Kaur, Sara Arredondo, Ankura Sitaula, Aref Zarin

**Affiliations:** 1 Biology Graduate Program, Texas A&M University, College Station, Texas, USA; 2 Department of Biology, Texas A&M University, College Station, Texas, USA; 3 Texas A&M Institute for Neuroscience, Texas A&M University, College Station, Texas, USA

**Keywords:** *Drosophila* larvae, neuromuscular junction remodeling, heterosynaptic structural plasticity, compensatory motor axon sprouting, ectopic muscle reinnervation, tonic and phasic motor neurons, locomotor recovery, neuron-subtype- and perturbation-specific responses

## Abstract

Motor neurons (MNs) form precise neuromuscular junctions (NMJs), but the extent to which individual MNs can reinnervate fully denervated muscles *in vivo* remains unclear. In the *Drosophila* larva, most muscles are co-innervated by a tonic (Ib) and a phasic (Is) MN. We show that when all tonic and phasic inputs to a dorsal muscle field are ablated, the surviving tonic MN1 undergoes robust heterosynaptic sprouting and forms ectopic, functional NMJs on fully denervated muscles. This plasticity is not induced by acute or chronic silencing and instead requires physical loss of neighboring MNs. Longitudinal imaging reveals that sprouting begins shortly after larval hatching and expands across development. In contrast, phasic MN-Is shows minimal remodeling within its partially innervated field. Quantitative analyses indicate that MN1 increases its synaptic territory only modestly and redistributes it across native and ectopic targets. These findings define neuron-subtype–specific, injury-dependent rewiring in an intact motor circuit.

## Highlights

• Complete muscle denervation induces sprouting of a bystander tonic motor neuron
• Bystander MN1 sprouting is triggered by loss but not silencing of nearby MNs
• Ectopic synapses restore muscle activity and partially rescue locomotion
• Sprouting begins early and expands across larval development

## Graphical abstract

**Figure:**
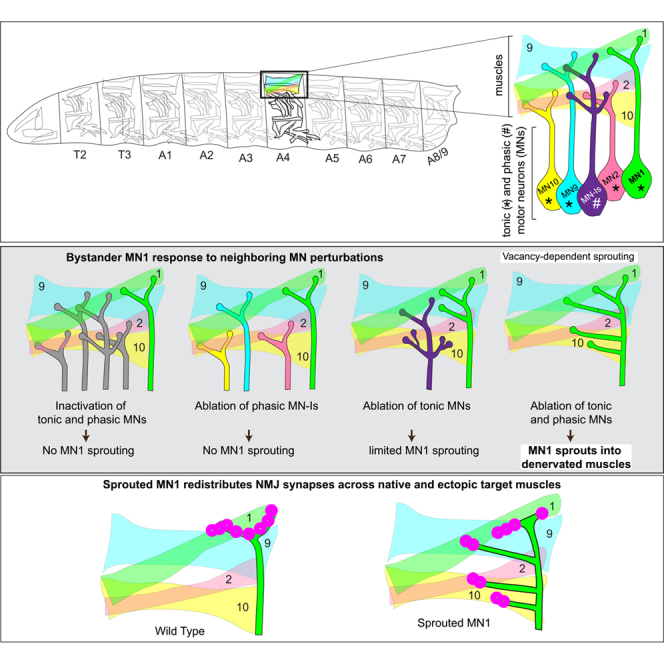

## Teaser

Neuroscience; Systems neuroscience

## Introduction

When a subset of neurons within a circuit is perturbed, the remaining non-perturbed—or *bystander*—neurons may undergo structural plasticity to compensate for the disruption and help restore circuit function. Neurons differ in intrinsic properties such as firing dynamics (for example, tonic versus phasic activity patterns), neurotransmitter identity, and synaptic architecture (such as large versus small boutons), yet it remains unclear whether all neuron types are equally capable of structural remodeling following neural perturbation. Moreover, whether the same bystander neuron exhibits similar or distinct plasticity responses to different forms of perturbation imposed on neighboring neurons—such as physical loss, disruption of action-potential generation, or blockade of spontaneous and/or evoked vesicle release—remains an open question. These gaps limit our ability to predict which neurons can compensate for circuit perturbations and under what conditions. To address these questions, we examined the capacity for axonal sprouting and neuromuscular-junction (NMJ) remodeling in physiologically distinct tonic and phasic motor neurons of *Drosophila* larvae subjected to multiple types of perturbation.

Classic vertebrate and invertebrate studies laid the foundation for understanding how surviving neurons respond to partial denervation. In adult rats, partial removal of a motor nerve’s axons was shown to cause the remaining fibers to sprout collateral branches that reinnervate denervated muscle fibers, resulting in enlarged motor units and hypertrophied residual axons.^1^^,^^2^ In mice, similar work demonstrated that collateral sprouting also occurs in mature motor neurons after partial muscle denervation, indicating that adult neurons retain a latent growth potential that can be reactivated by local cues from denervated targets.^3^ Parallel discoveries in invertebrates provided cellular and mechanistic detail. In leech, ablation of identified neurons was found to trigger selective reinnervation by homologous partners, restoring synaptic transmission and coordinated reflex output within the same segmental network.^4^^,^^5^ In crustaceans such as lobster, partial denervation of extensor muscles enhanced transmitter release and induced branch expansion of surviving axons within the same muscle field.^6^^,^^7^ In locusts, selective ablation of the fast extensor tibiae (FETi) neuron caused the slow extensor tibiae (SETi) neuron to form new NMJs on the previously fast-contracting muscle fibers, restoring fast contractions in these muscles.^8^ Together, these studies revealed a conserved capacity for heterosynaptic (cross neuronal) structural plasticity (HTSP)—a process through which surviving neurons detect and compensate for the loss of neighboring partners. Yet the cellular rules that determine which neurons remodel, and how their responses depend on the nature of the perturbation, remain largely unresolved. Here, we directly test this idea by comparing the remodeling responses of identified tonic and phasic motor neurons to distinct forms of perturbation, thereby defining the neuron-subtype-specific and perturbation-dependent rules that govern HTSP.

Due to its simplicity and powerful genetic toolkit, the *Drosophila* NMJ has long served as a leading model for dissecting conserved mechanisms of synaptic development and plasticity at single-cell resolution.^9^^,^^10^^,^^11^^,^^12^^,^^13^^,^^14^^,^^15^^,^^16^^,^^17^^,^^18^^,^^19^^,^^20^^,^^21^^,^^22^^,^^23^^,^^24^^,^^25^ Larval muscles are innervated by two classes of glutamatergic excitatory motor neurons (MNs): tonic-firing type Ib MNs, which form large boutons and typically innervate a single muscle, and phasic-firing type Is MNs, which form smaller boutons and innervate multiple muscles.^10^^,^^13^^,^^18^^,^^24^^,^^26^^,^^27^^,^^28^^,^^29^ These MNs converge on shared target muscles, creating a synaptic motif in which one tonic and one phasic MN co-innervate the same postsynaptic muscle. Such convergence of tonic and phasic inputs onto a common postsynaptic cell is an evolutionarily conserved circuit motif observed across both vertebrate and invertebrate systems.^30^^,^^31^^,^^32^

Previous work in *Drosophila* larvae has begun to reveal neuron-type-specific compensatory growth. Ablating—but not silencing—the phasic MN Is induces structural expansion of the NMJ formed by its co-innervating tonic partner MN1 on muscle 1, whereas loss of MN1 has no effect on MN Is.^18^^,^^24^^,^^26^^,^^27^ These findings suggest that tonic motor neurons are more structurally adaptive than phasic ones. Given that only one MN type at a time was ablated, the affected muscles retained partial excitatory input. Whether complete denervation evokes more robust or qualitatively distinct plastic responses—and which neurons are capable of compensating—remains unknown.

In this study, we compare structural compensation between identified tonic (Ib) and phasic (Is) motor neurons following physical ablation versus acute or chronic silencing of neighboring MNs. We show that the bystander tonic MN1 engages an ablation-dependent sprouting program that forms functional ectopic NMJs on a fully denervated dorsal muscle field, restoring muscle activation and improving peristaltic locomotion. This remodeling occurs within a broad developmental time window, revealing neuron-subtype-specific, and loss-dependent principles of heterosynaptic structural plasticity.

## Results

### Tonic MN1 sprouts and forms ectopic NMJs following complete denervation of neighboring muscles

**Figure 1:**
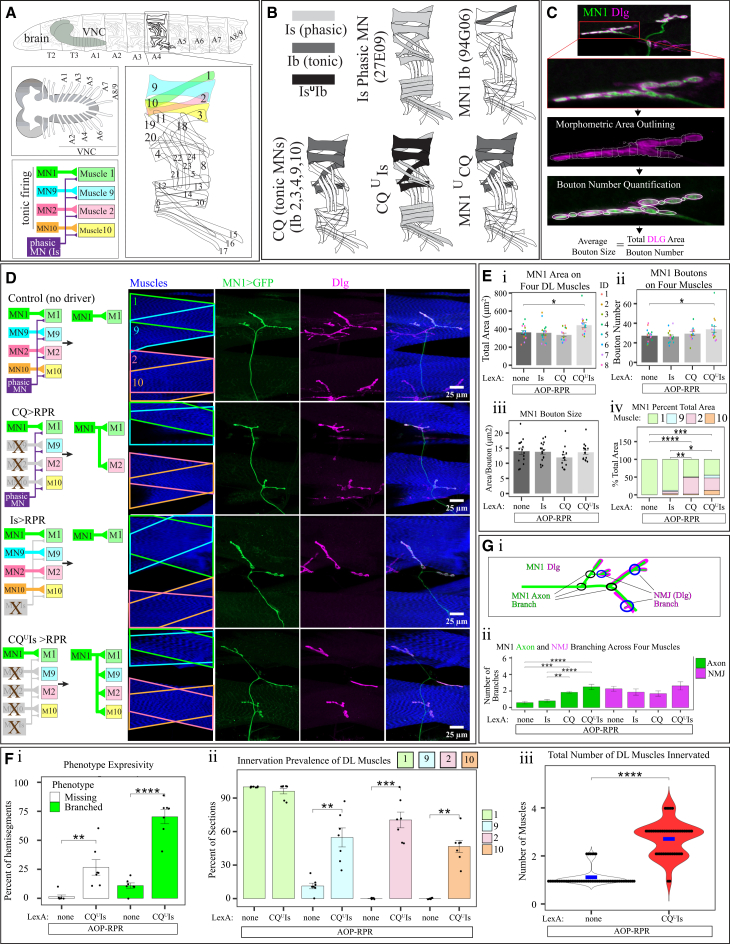
Tonic MN1 sprouts and forms ectopic NMJs following complete denervation of neighboring dorsal-longitudinal (DL) muscles (A) Schematics of the *Drosophila* larval CNS (brain + ventral nerve cord) and body-wall musculature. We analyzed four DL muscles (muscles 1, 2, 9, and 10), each normally co-innervated by a shared phasic glutamatergic MN-Is and distinct tonic Ib motor neurons (MN1, MN2, MN9, and MN10). (B) Target muscle coverage of Gal4 and LexA driver lines used for MN visualization or ablation. The union symbol (∪) denotes dual-driver lines. In MN-Is ∪ MN-CQ > RPR larvae, tonic, and phasic MNs innervating muscles 2, 3, 4, 9, and 10 are simultaneously ablated, producing complete excitatory denervation of nearby DL targets. (C) Workflow for NMJ imaging, segmentation, and quantitative morphometric analysis using an NMJ quantification pipeline adapted from prior work.^40^^,^^41^ (D) Representative immunostaining of MN1 axon morphology and NMJs. In controls, MN1 innervates only Muscle 1, and this pattern is unchanged after phasic MN-Is ablation (Is > RPR). Ablation of tonic MN2, MN9, and MN10 (CQ > RPR) induces MN1 sprouting selectively onto neighboring muscle 2. Co-ablation of tonic and phasic MNs (Is ∪ CQ > RPR) induces robust MN1 sprouting, forming ectopic NMJs on all denervated DL muscles (M2, M9, and M10) while retaining innervation of its native target (M1). Postsynaptic sites are labeled by discs large (Dlg, magenta); muscles are outlined using phalloidin (blue). MN1 was visualized using 94G06-Gal4 driving UAS-myr-GFP. Targeted MN ablation was achieved via LexA/OP-rpr/hid-mediated apoptosis. (E) Quantitative morphometric analysis of MN1 parameters. (1) Total MN1 surface area across DL muscles. Significant expansion occurs only in Is ∪ CQ > RPR larvae relative to control. LME: β = +79.6 ± 31.9 μm^2^; *p* = 0.0156. (2) Total bouton number. Significant increase only in the full-denervation condition. LME: β = +6.48 ± 2.61 boutons; *p* = 0.0161. Color-matched data points indicate hemisegments derived from the same larva. (3) Mean bouton size shows no significant differences across groups. (4) MN1 redistributes, rather than proportionally scales, presynaptic area after injury. In CQ > RPR larvae, MN1 forms ectopic NMJs only on muscle 2. In Is ∪ CQ > RPR larvae, MN1 co-innervates muscles 2, 9, and 10 in addition to muscle 1. In both CQ > RPR and Is ∪ CQ > RPR, the fraction of MN1 NMJ area on muscle 1 is significantly reduced compared to control. (F) Phenotypic prevalence and ectopic muscle acquisition analysis. (1) In Is ∪ CQ > RPR larvae, MN1 forms ectopic NMJs in 70.3% of segments or fails to reach any DL muscles in 26.6% of segments. Data points represent the percentage of analyzed hemisegments per larva. (2) Ectopic muscle choice frequency is significantly higher after dual MN ablation relative to controls, with muscle 2 being the most common non-native target (70.4% innervation of on muscle 2, 54.7% on muscle 9, and 46.6% on muscle 10). (3) MN1 commonly innervates three DL muscles per segment following full denervation. (G) Axon versus NMJ branchpoint analysis. (1) Branchpoint classification schematic (black: axon; blue: Dlg-positive NMJ). (2) Both CQ > RPR and Is ∪ CQ > RPR larvae show significantly increased axon branch points relative to control, while NMJ branch points remain unchanged, indicating that remodeling reflects *de novo* axonal sprouting rather than restructuring of existing NMJ arbor topology. Sample sizes: Control, Is > RPR, and Is ∪ CQ > RPR: *n* = 8 larvae (16 hemisegments). CQ > RPR: *n* = 7 larvae (13 hemisegments) for E, G. n = 7 (Control: 63 hemisegments, Is ∪ CQ > RPR: 66 hemisegments) for F. Data are represented by mean ± SEM. Statistics: LME for E (1) and (2) with larvae modeled as random intercepts to account for repeated hemisegment sampling. One-way ANOVA with Tukey HSD post-hoc test for E (3). Kruskal-Wallis with Dunn’s post-hoc test for G. Wilcoxon rank-sum tests for condition-pair comparisons in F. Significance: *p* < 0.05 (∗*), p < 0.01 (*∗∗*), p < 0.001 (*∗∗∗), *p* < 0.0001 (∗∗∗∗).

The *Drosophila* larval body comprises three thoracic segments (T1–T3) and nine abdominal segments (A1–A9). The central nervous system (CNS) consists of two brain lobes and a segmented ventral nerve cord (VNC), which functions analogously to the vertebrate spinal cord. Each VNC segment controls the muscles of its corresponding body segment (Figure 1A). In each abdominal segment, approximately 30 bilaterally symmetrical muscle pairs are innervated by 28 pairs of tonic and two pairs of phasic glutamatergic excitatory motor neurons (MNs) projecting from the segmental VNC^10^^,^^33^^,^^34^^,^^35^^,^^36^^,^^37^^,^^38^^,^^39^ (Figure 1A).

Here, we focused on the dorsal-longitudinal (DL) muscles—muscles 1, 2, 9, and 10—which receive convergent input from a shared phasic firing MN-Is (also known as RP2 or the common dorsal excitor, CDE) and four tonic (Ib) MNs: MN1, MN2, MN9, and MN10 (Figure 1A**)**.^10^^,^^33^^,^^34^^,^^35^^,^^36^^,^^37^^,^^38^^,^^39^ Previous work showed that apoptotic ablation of the phasic MN-Is leads to structural expansion of MN1, whereas MN1 ablation does not induce compensatory remodeling in MN-Is.^18^^,^^24^^,^^26^^,^^27^ Because those studies removed only one MN class at a time, leaving muscles *partially innervated*, it remained unclear how MN1 responds to completely denervated muscles located near its target muscle 1.

To address this, we genetically ablated either or both tonic and phasic MNs innervating muscles 2, 9, and 10 and examined structural remodeling by the bystander MN1 (Figures 1B–1D). For ablation, we used previously validated MN-LexA drivers^42^^,^^43^^,^^44^^,^^45^ to express the pro-apoptotic genes *reaper (rpr)* and *head involution defective (hid)*, inducing cell death in targeted MNs.^46^ The bystander MN1 axon was visualized using 94G06-Gal4 driving UAS-myr-GFP (hereafter referred to as MN1 > GFP). All analyses were performed on L3 larvae, focusing on abdominal segments A1–A7 only (thoracic segments were excluded). As larvae subjected to full MN ablation sometimes took one or two more days to reach L3, we controlled for this by collecting developmentally equivalent, size-matched 4-mm animals across all experimental groups.

MN1 did not form ectopic NMJs when only the phasic MN-Is was ablated (27E09-LexA > AOP-RPR, hereafter referred to as Is > RPR). When the tonic MNs innervating muscles 2, 9, and 10 were ablated (CQ-LexA > AOP-RPR, hereafter referred to as CQ > RPR), MN1 formed ectopic NMJs primarily on muscle 2 (Figures 1D and 1E). In striking contrast, simultaneous ablation of both tonic and phasic MNs (CQ ∪ Is > RPR) produced robust and widespread sprouting: MN1 generated ectopic branches in 70.3% of abdominal segments, whereas in 26.6% of segments it failed to project to any dorsal longitudinal muscles, including its native target muscle 1 (Figure 1F). In segments where sprouting occurred, MN1 co-innervated its native target (muscle 1) alongside one or more ectopic targets (muscles 2, 9, and 10). Among these, muscle 2 was the most frequently innervated ectopic muscle, with ectopic innervation observed in 70.4% of sprouting segments, compared to 54.7% for muscle 9 and 46.6% for muscle 10 (Figures 1D–1F).

To quantitatively assess how MN1 distributes its presynaptic territory across native and ectopic muscles, we implemented quantitative morphometric analysis^40^^,^^41^ to measure NMJ area and bouton number across these groups (Figure 1C). We used linear mixed-effects (LME) modeling, incorporating individual larvae as a random effect because multiple hemisegment measurements were obtained from each larva. LME analysis showed that MN1 total synaptic area increased significantly only in the complete-denervation condition (CQ ∪ Is > RPR), compared to controls (β = +79.6 ± 31.9 μm^2^; *p* = 0.0156). Neither phasic-only nor tonic-only ablation altered MN1 total area (*p* > 0.40), demonstrating that substantial expansion of MN1’s presynaptic domain occurs only when *all* co-innervating MNs are removed. Similarly, MN1’s total bouton number increased significantly only in the full-denervation group (β = +6.48 ± 2.61 boutons; *p* = 0.0161), with no change in the single-ablation groups (*p* > 0.45). Thus, MN1’s capacity to add boutons is likewise triggered exclusively by complete denervation.

Despite forming ectopic NMJs on up to three additional muscles, MN1 expanded its total presynaptic territory only modestly. This increase is far smaller than the ∼4-fold expansion that would be expected if MN1 were massively overgrowing to provide full input to three additional muscles, indicating that MN1 does not simply scale its total synaptic territory with target number. Instead, MN1 redistributed its synaptic investment: the proportion of total area allocated to its native muscle 1 decreased, and the remainder was partitioned among the newly innervated muscles (Figures 1D and 1E). These findings support the idea that MN1 operates under a constrained presynaptic “budget,” modestly increasing total synapse area under strong injury conditions but primarily compensating through redistribution rather than large-scale overgrowth.

Finally, analysis of axonal and NMJ branch points in the bystander MN1 revealed a significant increase in axonal branch points, consistent with injury-induced sprouting, in both CQ > RPR and CQ ∪ Is > RPR larvae. In contrast, Dlg-positive NMJ branch points showed no significant change, indicating that MN1 reaches ectopic, denervated dorsal-longitudinal muscles mainly through new axonal branching, which is subsequently followed by *de novo* NMJ formation on these targets (Figure 1G**)**.

Together, these results show that MN1 undergoes robust heterosynaptic sprouting only when both tonic and phasic excitatory MNs are eliminated and that functional reinnervation is supported by a combination of modest expansion and substantial redistribution of MN1’s presynaptic territory across denervated muscles.

### Phasic MN-Is retains its normal innervation pattern after loss of co-innervating tonic inputs

Our earlier experiments demonstrated that the tonic MN1 exhibits robust heterosynaptic sprouting when neighboring muscles are fully deprived of both tonic and phasic inputs. We next asked whether the phasic MN-Is possesses a comparable capacity for structural remodeling and NMJ expansion following the loss of its tonic co-innervators.

**Figure 2:**
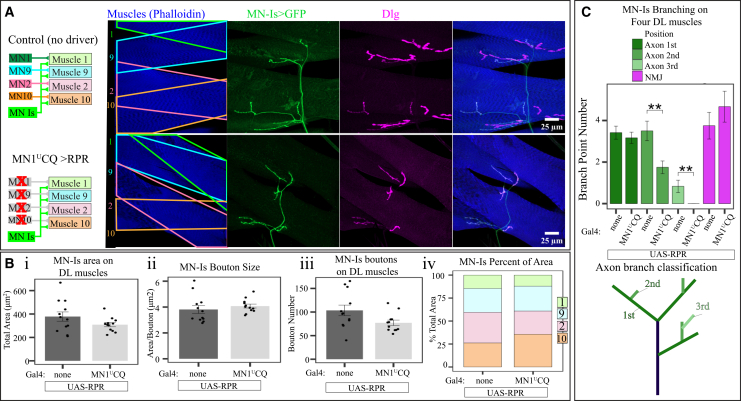
Phasic MN-Is retains its normal innervation pattern after loss of co-innervating tonic inputs (A) Representative NMJ immunostaining showing the bystander phasic MN-Is axon in control larvae and in larvae where all tonic Ib MNs innervating DL muscles (MN1, MN2, MN9, and MN10) were ablated (*MN1 ∪ CQ > RPR*). Postsynaptic sites are labeled with Dlg (magenta), muscles with phalloidin (blue), and MN-Is axons with myr-GFP driven by the 27E09 enhancer. Ablation of tonic MNs was achieved using the MN1-Gal4 ∪ CQ-Gal4/UAS-RPR system. (B) Quantification of Is NMJ parameters shows no significant differences in total NMJ area (1), bouton number (2), bouton size (3), or NMJ area distribution (4) between control and *MN1 ∪ CQ > RPR* larvae. Each data point represents a single hemisegment. (C) Ablation of tonic MNs results in a significant reduction in 2nd- and 3rd-order axonal branches of the bystander MN-Is. For both control and *MN1 ∪ CQ > RPR* groups, *n* = 6 larvae (12 hemisegments). Data points represent individual hemisegments. Data are represented by mean ± SEM. Statistical analysis: *t* test of pairs between conditions for B (1 and 4). Wilcox test of pairs between conditions B (2 and 3) and C (*p* < 0.05; ∗*p* < 0.01; ∗∗*p* < 0.001; ∗∗∗*p* < 0.0001).

Previous studies, in which only MN1 was ablated, reported no structural changes in NMJs formed by the phasic MN-Is on muscle 1.^18^^,^^24^^,^^26^^,^^27^ To extend these findings, we ablated additional tonic MNs (MN1, MN2, MN9, and MN10), thereby fully depriving the dorsal-longitudinal (DL) muscles 1, 2, 9, and 10 of all tonic innervations (Figure 2A). In both control and ablated groups, the phasic MN-Is was visualized using myr-GFP driven by an enhancer element cloned from the 27E09-Gal4 driver, which targets phasic MNs.

Following tonic MN ablation (CQ ∪ MN1 >RPR), the intact phasic MN-Is retained its normal innervation pattern on muscles 1, 2, 9, and 10 (Figure 2A). Despite the complete loss of tonic excitatory input, MN-Is did not exhibit notable structural changes in the NMJs it normally establishes on these muscles. Aside from a modest reduction in second- and third-order axonal branches in CQ ∪ MN1 > RPR larvae, bouton number, bouton area, and terminal size remained comparable between control and ablated groups (Figures 2B and 2C). These results suggest a difference in the structural plasticity potential of tonic versus phasic MNs.

However, it is important to note that in our paradigm the DL muscles remain innervated by MN-Is even after tonic MN ablation. As such, we could not determine whether MN-Is entirely lacks the capacity to sprout onto *non-native*, *fully denervated* muscles. Instead, our data show that MN-Is does not structurally respond to the loss of its tonic Ib partners and does not expand its normal innervation under conditions where it remains the sole surviving excitatory input. This interpretation is consistent with earlier work demonstrating the limited structural compensation of MN-Is following selective loss of MN1.^18^^,^^24^^,^^26^^,^^27^

### The bystander MN1 exhibits distinct structural responses to specific perturbations of neighboring MNs

To confirm that the robust sprouting observed in MN1 results specifically from the loss of neighboring motor neurons—and not from artifacts of the LexA/AOP genetic system—we repeated the ablation experiments using the Gal80^ts^/GAL4/UAS system. Gal80^ts^ inhibits GAL4 activity at 24°C but not at 29°C.^47^^,^^48^

**Figure 3:**
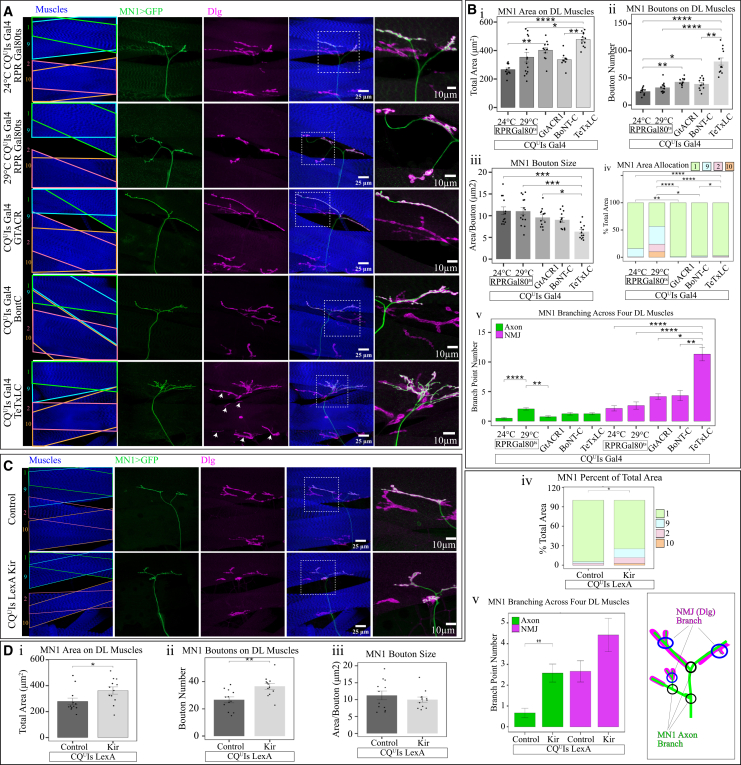
The bystander MN1 exhibits distinct structural responses to specific perturbations of neighboring MNs (A) Representative MN1 axon and NMJ morphology across genetic perturbations of co-innervating MNs. MN1 is visualized using MN1-LexA > AOP-myr-GFP. (B) Quantification of total MN1 NMJ area (1), total bouton number (2), and bouton size (3), MN1 NMJ allocation to different DL muscles (4), and MN1 axon branchpoint number (5), reflecting injury-induced or activity-dependent sprouting responses. Across conditions in A: in control larvae (CQ ∪ Is-Gal4/UAS-RPR, Gal80ts maintained at 24°C), MN1 displays normal axon projection pattern and native NMJ morphology. Genetic ablation of neighboring tonic and phasic excitatory MNs (CQ ∪ Is-Gal4/UAS-RPR, Gal80ts at 29°C) triggers extensive MN1 sprouting and *de novo* formation of ectopic NMJs on denervated DL muscles 2, 9, and 10. Acute optogenetic silencing of neighboring MNs (CQ ∪ Is-Gal4/UAS-GtACR1) elevates MN1 synapse area and bouton number relative to controls but does not induce ectopic innervation. Chronic silencing that blocks both evoked and spontaneous release (CQ ∪ Is-Gal4/UAS-BoNT-C) increases bouton number without ectopic muscle acquisition. Chronic silencing that selectively blocks only evoked synaptic release (CQ ∪ Is-Gal4/UAS-TeTxLC) results in strong increases in MN1 NMJ area, bouton number, and axon branch points compared to controls and all other perturbations. In this group, average MN1 bouton size is significantly reduced. Importantly, silenced presynaptic NMJ arbors themselves retain normal overall Dlg-positive NMJ scaffold morphology, indicating that MN1 remodeling occurs without large-scale structural degradation of the perturbed NMJs. (C) Representative images comparing MN1 morphology in control versus chronic silencing of neighboring MNs using the inward-rectifier potassium channel Kir2.1. (D) Quantification of total MN1 NMJ area (1), total bouton number (2), bouton size (3), MN1 NMJ allocation to different DL muscles (4) and MN1 axon branchpoint number (5). In D–F: MN1 is visualized using 94G06-Gal4 > UAS-myr-GFP. Chronic silencing of neighboring MNs with Kir2.1 was achieved using the LexA/OP system. In the CQ ∪ Is-LexA/AOP-Kir2.1 condition, the total area, bouton number and axon branching of MN1 are significantly increased, while the area on its native muscle 1 is decreased. Small ectopic synapses are formed on muscles 2, 9, and 10. Data points represent single hemisegments. Ablation group: *n* = 7 larvae (14 hemisegments). All other groups: *n* = 6 larvae (12 hemisegments). Data are represented by mean ± SEM. Statistical analysis (B): One-way ANOVA with Tukey HSD post-hoc test for B (1 and 3). Kruskal-Wallis with Dunn’s post-hoc test for B (2, 4, and 5). Statistical analysis (D): *t* test of pairs between conditions for D (1–3). Wilcox test of pairs between conditions for D (4 and 5). (∗*p* < 0.05; ∗∗*p* < 0.01; ∗∗∗*p* < 0.001; ∗∗∗∗*p* < 0.0001).

Using previously characterized CQ and Is GAL4 drivers,^26^^,^^39^^,^^42^^,^^44^^,^^49^^,^^50^ we targeted the tonic MN2, MN9, and MN10 along with the phasic MN-Is for UAS-reaper (rpr)-mediated ablation. MN1 was labeled with MN1-lexA >AOP-myr-GFP. Animals raised continuously at 29°C showed robust MN1 sprouting and ectopic NMJs on denervated muscles 2, 9, and 10, whereas controls maintained at 24°C (with functional Gal80 protein blocking GAL4 activity) showed no sprouting (Figures 3A, 3B, and S1). These findings confirm that MN1 sprouting is specifically triggered by the physical loss of neighboring MNs rather than by potential genetic artifacts.

We next tested whether silencing the activity of neighboring MNs—without their physical removal—would be sufficient to induce MN1 sprouting. For acute silencing, we used the light-activated chloride channel GtACR1, which causes chloride influx and neuronal hyperpolarization upon illumination.^51^^,^^52^ Previous work has demonstrated the efficacy of GtACR1 in silencing MNs and other neuronal subsets in *Drosophila* larvae.^44^^,^^45^^,^^49^^,^^53^ CQ ∪ Is > GtACR1 embryos and larvae were raised entirely in darkness to prevent developmental activation of the channel and were exposed to light only during a brief interval immediately prior to dissection. Under these conditions, MN1 maintained its normal innervation of muscle 1 and did not form ectopic NMJs on muscles 2, 9, or 10. We observed a modest increase in NMJ area and bouton number on muscle 1 relative to controls, with no change in bouton size (Figures 3A, 3B, and S1). This structural change is unlikely to arise from the brief light exposure immediately prior to dissection, suggesting that it reflects a longer-term consequence of altered neuronal activity, potentially arising from sustained effects of GtACR1 expression under these conditions, rather than an acute effect of illumination. Importantly, this phenotype is qualitatively distinct from the robust ectopic sprouting observed following neuronal ablation.

We then tested chronic silencing using botulinum neurotoxin-C (BoNT-C), which blocks both evoked and spontaneous neurotransmitter release.^22^^,^^23^ In CQ ∪ Is >BoNT-C larvae, MN1 did not form ectopic NMJs, but the number of boutons on muscle 1 was significantly increased relative to controls, despite no changes in NMJ area or bouton size (Figures 3A and 3B; Figure S1).

We then expressed tetanus toxin light chain (TeTxLC), which blocks evoked but not spontaneous neurotransmitter release.^54^ In CQ ∪ Is >TeTxLC larvae, MN1 again did not form ectopic NMJs, but showed substantial NMJ expansion on its native target, muscle 1 (Figure 3A–3C; Figure S1). This included significantly increased NMJ area, branching, and bouton number, along with a marked reduction in average bouton size (Figure 3B and 3C; Figure S1). These features are consistent with satellite bouton formation previously observed following TeTxLC-mediated silencing of MN-Is.^18^^,^^26^ Notably, Dlg staining revealed that the NMJs of TeTxLC-silenced tonic MNs maintained normal morphology and did not exhibit the extensive NMJ overgrowth observed in the bystander MN1 (Figure 3A).

Finally, we asked whether chronic suppression of excitability—rather than neurotransmission—could influence the bystander MN1. To do this, we expressed the inwardly rectifying potassium channel Kir2.1^55^^,^^56^ in CQ ∪ Is MNs using the lexA/OP system while visualizing MN1 with GFP (MN1 > GFP). Kir2.1 expression is widely used in *Drosophila* to hyperpolarize neurons and strongly suppress their firing. In these larvae, we observed mild sprouting of MN1 onto muscles 2, 9, and 10 (Figures 3D and 3E). Notably, inward-rectifier potassium channels have been shown in other systems to silence neurons and, in some cases, induce neuronal stress or apoptosis when strongly overexpressed—such as the ROMK1/Kir1.1 channel, which triggers chronic silencing and apoptotic degeneration in cultured hippocampal neurons.^57^ Thus, the mild MN1 sprouting observed here may reflect the incomplete apoptosis of the Kir2.1-expressing MNs rather than a pure consequence of reduced excitability. These results are consistent with a scenario in which MN1 responds to compromised or degenerating neighboring MNs.

Taken together, these experiments demonstrate that robust heterosynaptic sprouting of MN1 requires the physical ablation of neighboring MNs. Silencing neighboring MNs—whether acute or chronic—does not induce ectopic sprouting, although it can modulate the morphology of MN1’s native NMJ. Only perturbations that involve actual loss or degeneration of neighboring MNs produce the strong sprouting response observed following ablation.

### Ectopic MN1 sprouting restores muscle activation and partially rescues crawling behavior

Thus far, we have shown that concurrent ablation—but not silencing—of neighboring tonic and phasic MNs triggers robust sprouting of the bystander MN1, resulting in ectopic NMJs on fully denervated muscles 2, 9, and 10. To determine whether these ectopic NMJs are functional and capable of activating the muscles they innervate, we performed *in vivo* muscle calcium imaging in intact, forward-crawling larvae.

**Figure 4:**
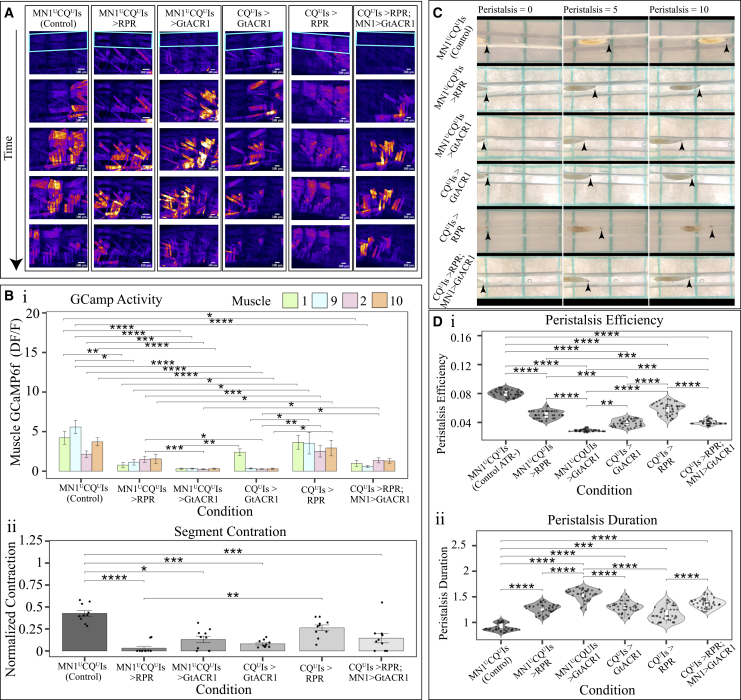
Ectopic MN1 sprouting restores muscle activation and partially rescues crawling behavior (A) Muscle calcium imaging in intact, forward-crawling larvae of different genotypes. Each column shows still images from three adjacent abdominal segments at successive time points during a forward peristaltic wave. GCaMP6f fluorescence (expressed under the 44H10 muscle-specific enhancer) increases during muscle contraction. Cyan rectangles mark dorsal-longitudinal (DL) muscles in the top images. In control larvae (MN1 ∪ CQ ∪ Is-lexA/Aop-GTACR ATR [−]), DL muscles contract sequentially, with GCaMP6f signals propagating from posterior (right) to anterior (left) during crawling. Ablation of all tonic and phasic MNs (MN1 ∪ CQ ∪ Is-lexA/Aop-RPR) abolishes DL muscle activity, leaving ventral and lateral muscles unaffected. Similarly, acute silencing of these MNs (MN1 ∪ CQ ∪ Is-lexA/Aop-GtACR1 ATR [+]) eliminates DL muscle activity. In CQ ∪ Is-lexA/Aop-GtACR1 ATR (+) larvae, only Muscle 1 remains active, consistent with preserved MN1 input. In contrast, CQ ∪ Is-lexA/Aop-RPR larvae exhibit robust calcium activity and contractions in all four DL muscles, indicating functional reinnervation by sprouted MN1. Acute silencing of the sprouted MN1 in CQ ∪ Is-lexA/Aop-RPR; MN1-Gal4>UAS-GtACR1 ATR (+) larvae abolishes DL muscle activity. Frames are taken from Videos S1, S2, S3, S4, S5, S6 and S7. (B) Quantification of muscle calcium imaging seen in (A). (1) GCaMP6f signal in DL muscles (1, 2, 9, and 10) confirms observations in (A). (2) Muscle contraction of single hemisegments. All groups except CQ ∪ Is-lexA/Aop-RPR exhibit significantly less contraction compared to control. And CQ ∪ Is-lexA/Aop-RPR contracts significantly more than MN1 ∪ CQ ∪ Is-lexA/Aop-RPR. *n* = 5 larvae per genotype (10 hemisegments total). Statistical analysis: Kruskal-Wallis test with Dunn’s post-hoc correction (∗*p* < 0.05, ∗∗*p* < 0.01, ∗∗∗*p* < 0.001, ∗∗∗∗*p* < 0.0001). (C) Representative crawling trajectories of larvae from the genotypes in (A). Each row shows larval positions at the start line (peristalsis = 0), after 5 peristalses, and after 10 peristalses. Among all groups, control larvae (MN1 ∪ CQ ∪ Is-lexA/+) ranked first in distance traveled, followed by CQ ∪ Is-lexA/Aop-RPR larvae, which ranked second. In contrast, MN1 ∪ CQ ∪ Is-lexA/Aop-GtACR1 larvae showed the least forward progression. (D) Quantification of peristalsis efficiency (1) (distance traveled per peristaltic wave) and peristalsis duration (2) (time required to complete a wave). Control larvae (MN1 ∪ CQ ∪ Is-lexA/+) exhibited the highest peristalsis efficiency and shortest duration, followed by CQ ∪ Is-lexA/Aop-RPR larvae. Silencing the sprouted MN1 in CQ ∪ Is-lexA/Aop-RPR; MN1-Gal4>UAS-GtACR1 larvae reduced locomotor performance to levels comparable with other two silenced groups. *n* = 40 larvae per group, with each dot representing an individual animal. Data are represented by mean ± SEM. Statistical analysis: Kruskal-Wallis with Dunn’s post-hoc test D (1). One-way ANOVA with Tukey HSD post-hoc test for D (2). (∗*p* < 0.05, ∗∗*p* < 0.01, ∗∗∗*p* < 0.001, ∗∗∗∗*p* < 0.0001).

As a baseline, we first examined muscle calcium activity in larvae where all tonic and phasic MNs innervating dorsal muscles 1, 2, 9, and 10 were either genetically ablated (CQ-lexA ∪ Is-lexA ∪ MN1-lexA > RPR) or acutely silenced (CQ-lexA ∪ Is-lexA ∪ MN1-lexA > GtACR1) (Figures 4A–4C). In both groups, the dorsal-longitudinal (DL) muscles failed to show calcium transients or contraction during forward crawling, confirming complete loss of excitatory input. In contrast, ventral and lateral muscles with intact MN innervation exhibited robust calcium activity and contractions (Figure 4A and 4B; Videos S1, S2, and S3). These observations validate that (1) both GtACR1-mediated silencing and RPR-mediated ablation effectively eliminate MN output and (2) the MN driver lines selectively target the intended MNs without affecting off-target MNs.

Document S1. Figures S1–S3 and supplemental references

Table S2. Staining reagents Table

Video S1. Control larva exhibits coordinated DL muscle activity during crawling In an unperturbed animal (MN1 ∪ CQ ∪ Is-lexA > Aop-GtACR1, ATR–), GCaMP6f fluorescence reveals robust and sequential activation of dorsal longitudinal (DL) muscles.
Calcium signals initiate in posterior segments and propagate anteriorly, reflecting normal peristaltic waves during forward locomotion.

We then repeated these experiments but spared MN1. In the acute silencing group (CQ-lexA ∪ Is-lexA > GtACR1), only muscle 1 displayed calcium transients during crawling, consistent with MN1 maintaining its normal innervation (Figure 4A and 4B; Video S4). By contrast, in the ablation group (CQ-lexA ∪ Is-lexA > RPR), all four dorsal muscles (1, 2, 9, and 10) exhibited muscle calcium transients and contraction, demonstrating that ectopic NMJs formed by MN1 are capable of restoring contractile activity in denervated muscles (Figure 4A and 4B; Video S5). Consistent with restored muscle calcium transients and contractions, neural calcium (GCaMP6f) imaging of sprouted MN1 axon terminals confirmed that MN1 fires in phase with larval segment movement (Video S6).

Video S2. Complete ablation of DL-innervating MNs abolishes DL muscle activity In larvae with all tonic and phasic DL-innervating MNs ablated (MN1 ∪ CQ ∪ Is-lexA > Aop-RPR), DL muscle contractions and GCaMP6f signals are completely absent, confirming effective denervation.
Activity in ventral and lateral muscles remains intact, highlighting lexA-driver specificity.

Video S3. Acute silencing of all DL-innervating MNs eliminates DL muscle activity Following light-dependent activation of GtACR1 in MN1, CQ, and Is neurons (MN1 ∪ CQ ∪ Is-lexA > Aop-GtACR1, ATR+), DL muscle calcium activity is entirely suppressed.
This confirms the specificity of MN-lexA drivers and effectiveness of GtACR1 in silencing the MNs of interest.

Video S4. Only Muscle 1 remains active when MN1 is spared from silencing In CQ ∪ Is-lexA > Aop-GtACR1, ATR+ larvae (MN1 spared), calcium activity is preserved exclusively in Muscle 1, MN1’s native target.
DL muscles innervated by silenced MNs remain inactive, providing functional validation of MN1’s exclusive input to its native muscle-1.

To directly test whether the sprouted MN1 drives these responses, we acutely silenced MN1 (MN1-Gal4 >GtACR1) in larvae where neighboring MNs had been ablated (CQ-lexA ∪ Is-lexA > RPR). Silencing MN1 abolished calcium activity in all four dorsal muscles during crawling, confirming that the sprouted MN1 terminals are functionally active and required for muscle contraction (Figure 4A and 4B; Video S7).

Video S5. MN1-driven ectopic NMJs restore DL muscle activity following ablation In CQ ∪ Is-lexA > Aop-RPR animals, where all native DL-innervating MNs except MN1 are ablated, GCaMP6f imaging reveals restored activity across Muscles 1, 2, 9, and 10.
These data indicate that sprouted MN1 terminals form functional ectopic NMJs capable of driving muscle contractions.

Next, we investigated whether MN1 sprouting contributes to behavioral recovery. We quantified forward crawling by measuring peristalsis efficiency (distance traveled per peristalsis) and peristalsis duration (time to complete a forward wave). Control larvae displayed a peristalsis efficiency of ∼0.8 mm per wave. Larvae lacking dorsal MN inputs (CQ-lexA ∪ Is-lexA ∪ MN1-lexA > RPR) showed markedly reduced efficiency, but those with intact MN1 (CQ-lexA ∪ Is-lexA > RPR) performed significantly better, indicating partial restoration of crawling (Figure 4D and 4E; Video S8).

Video S6. The sprouted motor axons and NMJs of bystander MN1 show GCaMP6f activity Genotype: CQ ∪ Is-lexA > Aop-RPR; MN1-Gal4 > UAS-GCaMP6f.

Notably, CQ-lexA ∪ Is-lexA > RPR larvae also outperformed CQ-lexA ∪ Is-lexA > GtACR1 larvae (Figure 4D and 4E; Video S8), suggesting that ablation-induced sprouting of MN1 (occurring in the ablated group but not in the silenced group) underlies this improvement. However, because Is-lexA also targets ventral-projecting phasic MNs, it is possible that structural plasticity of ventral tonic MNs contributed to recovery. To isolate MN1’s contribution, we silenced the sprouted MN1 in CQ-lexA ∪ Is-lexA > RPR larvae using MN1-Gal4 >GtACR1. This manipulation significantly reduced peristalsis efficiency compared to unsilenced CQ-lexA ∪ Is-lexA > RPR larvae (Figure 4D and 4E; Video S8), directly demonstrating that MN1 sprouting is adaptive and improves locomotion.

Across all genotypes, we observed an inverse relationship between peristalsis efficiency and peristalsis duration: larvae traveling shorter distances per peristaltic wave also exhibited slower propagation of contractions across body segments, resulting in longer peristalsis durations (Figure 4E; Video S8).

Together, these results establish that MN1 ectopic sprouting restores muscle contractility and partially rescues motor behavior, highlighting its role as a compensatory response following injury.

### MN1 sprouting initiates early and expands progressively across larval stages

In *Drosophila*, embryogenesis lasts ∼22–24 h at 25°C, after which the embryo hatches into a first instar larva (L1) capable of foraging, feeding, and performing locomotor behaviors such as forward crawling. By hatching, core motor circuits are already established, with motor neurons forming functional synapses onto their target muscles. The L1 stage lasts ∼25 h, followed by molting into second (L2) and third instar (L3) stages, each lasting ∼24–25 h before pupation. Our initial characterization of MN1 heterosynaptic sprouting was performed in 4-mm-long L3 larvae across different groups. To determine when sprouting begins and how it evolves across development, we monitored MN1 axons longitudinally in animals where neighboring tonic and phasic MNs were genetically ablated.

**Figure 5:**
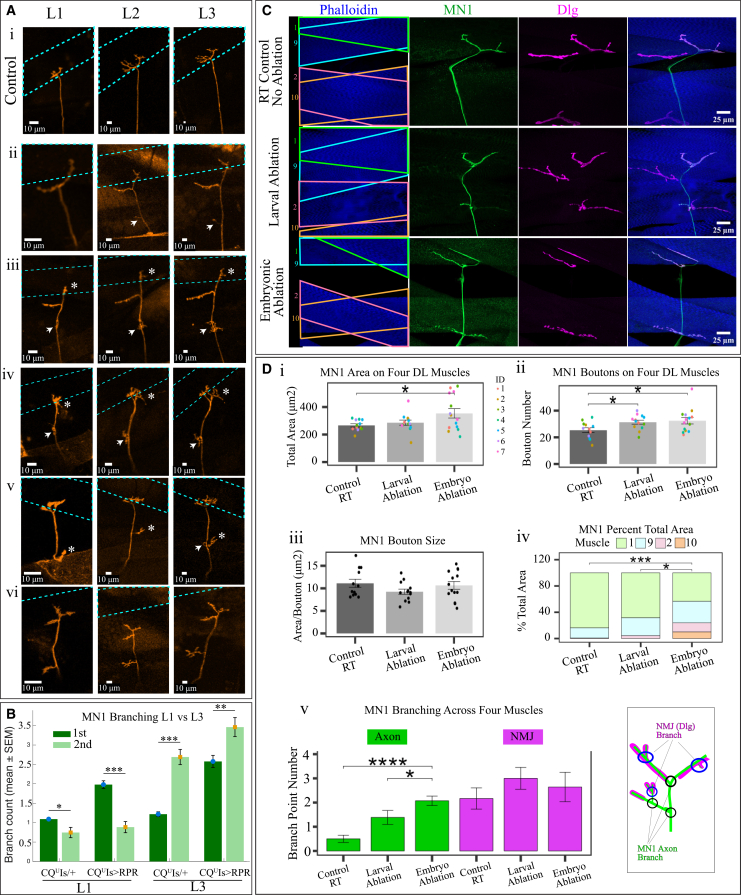
MN1 sprouting initiates early and expands progressively across larval development (A) Longitudinal *in-vivo* imaging of MN1tdTomato in the same abdominal hemisegment from L1 to wandering L3. In controls (CQ ∪ Is-Gal4/+; MN1-LexA > AOP-tdTomato), MN1 innervates only its native target muscle 1 at L1 and elaborates a T-shaped dorsal-longitudinal arbor by L3. Apoptotic ablation of neighboring excitatory MNs (CQ ∪ Is-Gal4/UAS-RPR; MN1-LexA > AOP-tdTomato) induces early axonal buds that grow substantially by L3, occasionally acquiring innervation of other nearby denervated DL muscles (2, 9, 10), or in rare cases, bypassing Muscle 1 entirely. (B) MN1 axon branch complexity quantified longitudinally from L1 to wandering L3. Developmental and genotype effects on branch order were analyzed using a linear mixed-effects model with fixed effects for stage (L1, L3), genotype (control, ablated), and their interaction, and each larva as a random intercept. Blue circles (first-order) and orange squares (second-order) represent LME model-predicted marginal means overlaid on the empirical barplot. First-order branches showed a strong genotype main effect (*p* = 4.37 × 10^−10^, LME) and a significant genotype × stage interaction (*p* = 0.0155, LME), indicating that L1→L3 first-order sprouting depends on neighboring MN ablation. Second-order branches exhibited a robust stage main effect across genotypes (*p* = 7.07 × 10^−13^, LME) but no significant stage × genotype interaction, meaning the developmental increase from L1→L3 was similar in control and ablated larvae. Within each stage, first-versus second-order branch investment was compared using paired two-group tests (L1 control *p* = 0.0188; L1 ablated *p* = 5.12 × 10^−7^; L3 control *p* = 1.84 × 10^−9^; L3 ablated *p* = 0.0045; paired *t* tests). *n* = 5 larvae (control: 57 hemisegments, CQ U Is > RPR: 54 hemisegments). (C) Temporally restricted larval denervation using Gal80ˆts (24 °C → 29 °C at hatching) induces detectable MN1 sprouting, but at reduced magnitude relative to continuous 29 °C embryonic-onset denervation. No sprouting was seen in 24 °C controls. (D) Quantification of MN1 native versus ectopic NMJ growth following temporally controlled denervation. (1 and 2) Linear mixed-effects (LME) analysis revealed a strong dependence on the developmental timing of denervation. (1) Relative to controls, larval-only ablation did not significantly increase total MN1 synaptic area (LME β = +13.63 ± 32.60 SE, *p* = 0.678), whereas embryonic-onset ablation produced a robust and significant expansion (LME β = +79.99 ± 32.15 SE, *p* = 0.0176). (2) Bouton number showed a different pattern: larval-only ablation caused a significant increase (LME β = +5.97 ± 2.74 SE, *p* = 0.0358), and embryonic ablation produced an even larger increase (LME β = +7.10 ± 2.69 SE, *p* = 0.0123). (3) Average bouton size (area/bouton number) did not show a significant difference across groups. (4) Proportional distribution of MN1 NMJ area on individual DL muscles. (5) MN1 Axon and NMJ branching across different groups. Both embryonic and larval ablation show a higher number of MN1 axon branches (but not NMJ branches). Data are represented by mean ± SEM. One-way ANOVA with Tukey HSD post-hoc test for D (3). Kruskal-Wallis with Dunn’s post-hoc test for D (4 and 5). (∗*p* < 0.05; ∗∗*p* < 0.01; ∗∗∗*p* < 0.001; ∗∗∗∗*p* < 0.0001). Data points in 1–3 represent single hemisegments. Control group: *n* = 6 larvae (12 hemisegments). For embryonic ablation: *n* = 7 larvae (14 hemisegments). Embryonic and control datasets are the same as in Figure 3. For larval ablation, *n* = 7 larvae (13 hemisegments).

Using live imaging of MN1 labeled with tdTomato, we tracked the same hemisegments at L1 and L3 and quantified 1st-, 2nd-, and 3rd-order axon branches. Between imaging sessions, larvae were recovered and returned to food to continue development. This allowed us to quantify and distinguish 1st-, 2nd-, and 3rd-order axon branches extending from MN1’s main shaft (Figure 5A).

At L1, MN1 axons in ablated larvae already showed more 1st-order branches than controls, indicating that sprouting is initiated very early after denervation. By L3, the difference between ablated and control larvae increased further. Linear mixed-effects (LME) analysis—which accounts for repeated measurements from the same hemisegment—confirmed that the increase in 1st-order branches from L1 to L3 was significantly larger in ablated animals than in controls (*p* = 0.00319), demonstrating that loss of neighboring motor neurons accelerates the developmental expansion of MN1 sprouting. For 2nd-order branches, both groups increased from L1 to L3, but ablated larvae displayed a markedly greater rise (*p* < 10^−13^), consistent with progressive amplification of ectopic growth. Third-order branches were rare at L1 but increased modestly by L3 in both groups. (Figures 5A and 5B). Together, these repeated-measures results indicate that MN1 sprouting begins shortly after denervation but expands substantially across the L1→L3 transition, revealing the larval period as a major developmental window for heterosynaptic plasticity.

At the earliest larval stage (L1), MN1 axons already initiated sprouting following full excitatory denervation, with ablated larvae exhibiting significantly elevated first-order branching relative to controls and showing a pronounced developmental expansion by wandering L3. Longitudinal L1→L3 changes were evaluated using paired t-tests on matched hemisegments from the same larvae, and genotype-by-developmental dependencies were further tested using an LME including LarvaID as a random intercept (Figure 5B**)**.This analysis revealed that first-order sprouting differed significantly by genotype across development (genotype × stage interaction *p* = 0.0155), second-order complexity increased strongly by developmental stage independent of genotype (*p* = 7.07 × 10^−13^), and third-order branching rose modestly across larval development without interaction (*p* = 5.28 × 10^−5^), identifying the L1→L3 larval period as a key window for heterosynaptic structural plasticity (Figure 5B**)**.

To directly test whether sprouting can be induced exclusively during larval stages, we performed temporally restricted MN ablation using Gal80^ts^,^47^^,^^48^ which suppresses Gal4 activity at 24 °C and permits rpr/hid-mediated cell death at 29 °C. For larval-only ablation, CQ-Gal4 ∪ Is-Gal4 >UAS-RPR, Gal80^ts^ embryos were maintained at 24 °C to prevent embryonic ablation and shifted to 29 °C from L1 through L3. As reference groups, we included (1) continuous 24 °C controls (Gal80^ts^ active; no ablation) and (2) continuous 29 °C ablation animals, in which MN death begins during embryogenesis. Larval-only ablation resulted in clear but modest MN1 sprouting, with ectopic NMJs present on denervated muscles (Figures 5C and 5D**)**.

Linear mixed-effects (LME) analysis revealed a strong dependence on the developmental timing of denervation. Relative to controls, larval-only ablation did not significantly increase total MN1 synaptic area (β = +13.63 ± 32.60 SE, *p* = 0.678), whereas embryonic-onset ablation produced a robust and significant expansion (β = +79.99 ± 32.15 SE, *p* = 0.0176). Bouton number showed a different pattern: larval-only ablation caused a significant increase (β = +5.97 ± 2.74 SE, *p* = 0.0358), and embryonic ablation produced an even larger increase (β = +7.10 ± 2.69 SE, *p* = 0.0123). These data indicate that while MN1 retains the capacity to remodel when denervation occurs during larval life, full presynaptic expansion is achieved only when ablation begins during embryogenesis, revealing a developmental constraint on the magnitude of heterosynaptic sprouting (Figure 5D**)**.

Taken together, these results demonstrate that MN1 sprouting begins early but expands substantially throughout larval development, with the L1-L3 period representing a key window for injury-induced ectopic NMJ formation. The combination of longitudinal imaging and temporally restricted ablation positions larval development as the primary phase, during which MN1 detects denervation and engages its compensatory growth program.

## Discussion

### Neuron-type-specific heterosynaptic sprouting requires complete loss of presynaptic partners

Our study demonstrates a neuron-subtype-specific form of heterosynaptic structural plasticity in the *Drosophila* larval motor system. When both tonic and phasic motor neurons innervating the dorsal longitudinal muscle field are ablated, the surviving tonic MN1 undergoes robust axonal sprouting and forms ectopic NMJs on multiple denervated muscles that it does not normally target. These ectopic terminals restore calcium responses in previously denervated muscles and partially rescue crawling, indicating that the remodeling is functionally meaningful. In contrast, neither the phasic MN-Is nor MN1 itself exhibited comparable sprouting when neighboring neurons were chronically or acutely silenced, rather than eliminated. Thus, complete physical loss of presynaptic partners—not reduced activity—is required to evoke this form of plasticity.

This requirement for full presynaptic loss is consistent with decades of classical work across vertebrates and invertebrates. In vertebrates, partial denervation induces collateral sprouting from surviving motor axons that reoccupy vacant postsynaptic territories and expand the remaining motor units.^1^^,^^2^^,^^3^^,^^58^ In the leech, ablation of an annulus erector (AE) motor neuron leads to compensatory sprouting exclusively from the remaining AE partner—either the anterior or posterior AE—while non-homologous motor neurons remain structurally stable despite proximity.^4^^,^^5^ In crustaceans, partial denervation of extensor muscles strengthens release and expands arbors of surviving axons sharing the same target field, with no response from unrelated neurons.^6^^,^^7^ In locusts, ablation of the fast extensor tibiae (FETi) neuron induces sprouting from the slow SETi neuron onto normally fast muscle fibers, restoring fast contractions—even though the complementary manipulation (testing FETi sprouting after SETi removal) was not performed.^8^ Across these diverse systems, denervated targets emit growth-permissive cues, neuron identity determines which cells can respond, and compensatory innervation can restore function even in non-native postsynaptic territories. Our results extend these principles to a well-defined tonic-phasic motor pair in *Drosophila* and show that complete presynaptic elimination is necessary to reveal the full sprouting capacity of tonic MNs.

### A fully denervated muscle field reveals MN1’s hidden sprouting capacity

Recent *Drosophila* studies showed that tonic Ib MNs—but not phasic Is MNs—exhibit structural expansion following partial loss of co-innervating partners.^18^^,^^24^^,^^26^^,^^27^ However, in these studies the muscle remained partially innervated, because either the tonic or phasic neuron was ablated, but not both. For example, ablating—but not silencing—the phasic MN-Is produces structural expansion of MN1 on muscle 1, yet the muscle still receives input from MN2, MN9, and MN10. Thus, earlier studies could not determine whether a surviving MN could sprout to fully denervated muscles.

By simultaneously ablating all tonic and phasic MNs co-innervating the dorsal longitudinal muscles, we created a completely denervated muscle field and uncovered a previously unrecognized capacity for MN1 to form ectopic NMJs on multiple non-native muscles and partially restore crawling. This paradigm shows that even a single intact input can suppress sprouting, and that MN1’s full growth potential becomes apparent only when the postsynaptic field is entirely vacated. This mirrors classic findings in leech AE MNs,^4^^,^^5^ *Drosophila* larval MNs,^59^ and crustacean extensor MNs,^6^^,^^7^ where sprouting occurs only when the target field is substantially or completely denervated.

Importantly, our work shows that MN1’s sprouting capacity is intrinsically limited. Although MN1 forms ectopic terminals on multiple denervated muscles, the total synaptic area and bouton number rise only modestly, consistent with an upper bound on the neuron’s ability to expand its presynaptic output.

### Determinants of sprouting patterns: Muscle accessibility, neighboring neuron integrity, and intrinsic MN identity

The spatial organization of the dorsal longitudinal muscle group likely contributes to the pattern of MN1 reinnervation. Muscles 1 and 2 form the internal layer of the group, whereas muscles 9 and 10 lie externally, overlying muscles 2 and 10. Along the dorsoventral axis, muscle 1 sits above muscle 2, and muscle 9 sits above muscle 10.^39^^,^^60^ In ablated larvae, the sprouting-competent MN1 would be expected to encounter muscle 2 first upon entering the muscle field, while reaching the externally positioned muscles 9 and 10 requires additional lateral projection. This anatomy likely contributes to the more frequent reinnervation of muscle 2. Interestingly, despite being less frequently reinnervated, muscle 9 showed *stronger* calcium recovery than muscle 2, indicating that functional rescue does not scale linearly with anatomical extent and may depend on postsynaptic or architectural specializations.

Silencing of neighboring MNs—whether acute (GtACR1) or chronic (BoNT-C, TeTxLC)—did not induce ectopic sprouting, although increases in bouton number or NMJ area occurred at MN1’s native synapse. Thus, reduced neurotransmission or impaired spiking alone is insufficient to drive heterosynaptic sprouting. Our Kir2.1 experiments further support this conclusion. Chronic Kir2.1 expression strongly hyperpolarizes neurons. However, overexpression of potassium channels such as Kir1.1 and kv2.1 has also been reported to cause cellular stress and even apoptosis.^57^^,^^61^ Therefore, the mild MN1 sprouting we observed under Kir2.1 perturbation could be a response to physiological stress in neighboring MNs, rather than a consequence of reduced excitability alone. Taken together, these data indicate that robust heterosynaptic sprouting requires the loss or functional compromise of presynaptic partners, not inactivity per se.

A final consideration is that tonic and phasic MNs differ intrinsically in ways that may influence their remodeling potential. Tonic MN1 exhibits larger boutons, more expansive active zones, lower baseline release probability, and elevated expression of growth-promoting genes such as Wnt4, Pyramus, Toll-6, and SiaT. Conversely, phasic MN-Is displays compact active zones, high initial release probability, dense VGCC clustering, and elevated calcium-buffering proteins such as Cbp53E.^22^^,^^23^^,^^30^^,^^42^^,^^62^^,^^63^ These structural and molecular distinctions likely make tonic MNs more growth-competent, while phasic MNs are optimized for rapid, high-fidelity transmission—properties that may naturally limit their ability to undergo structural expansion.

### Differences in plasticity capacity between mature and immature neurons

The magnitude of sprouting is also shaped by developmental context. Across systems, immature neurons exhibit considerably greater growth potential than mature ones. In locusts, SETi compensates for FETi loss during larval stages, whereas the same lesion immediately after adult molting fails to induce sprouting and instead leads to muscle degeneration.^8^ In leech embryos, interneuron ablation provokes extensive compensatory growth, while the same injury in adults yields persistent functional deficits.^64^ Such patterns reflect a general reduction in neuronal growth potential across maturation due to intrinsic changes in gene expression, cytoskeletal dynamics, and signaling pathways.^65^

*Drosophila* follows similar rules. Embryonic MN ablation generates widespread sprouting across several muscle fields, whereas ablation shortly after larval hatching produces more spatially confined reinnervation. Although larval MNs retain substantial growth competence compared with mature vertebrate CNS neurons, their remodeling capacity is nonetheless constrained by developmental stage and by the local permissiveness of the muscle environment.

### Limitations of the study

#### Caveats in assessing plasticity in the phasic MN-Is

Although MN-Is does not expand its normal innervation following the loss of tonic Ib partners, in our paradigm the dorsal longitudinal muscles remain innervated by MN-Is itself. Thus, we cannot conclude that MN-Is inherently lacks the capacity to sprout onto fully denervated, non-native muscles. Instead, our data show that MN-Is does not expand its established innervation when it is the sole surviving excitatory input within its muscle field. Attempts to test phasic sprouting on a truly denervated target—such as muscle 11, which normally lacks MN-Is input—were not feasible because Exex-Gal4, the only driver labeling its tonic partner, also targets many additional MNs, and driving apoptotic genes under this driver caused embryonic lethality. For this reason, conclusions regarding MN-Is plasticity must remain appropriately bounded.

## Resource availability

### Lead contact

Requests for further information and resources should be directed to and will be fulfilled by the lead contact, Aref Zarin (azarin@bio.tamu.edu).

### Materials availability

All fly lines generated and/or used in this study are available from the lead contact upon request.

### Data and code availability

All the raw data along with MATLAB and R codes used for data analysis and visualization have been deposited to Mendeley Data repository which can be accessed here: "Mendeley Data: https://data.mendeley.com/datasets/c9fdgs69xx/2".

## Acknowledgments

We are grateful to the Bloomington Drosophila Stock Center, Chris Doe, Troy Littleton, Dion Dickman, Keiko Hirono, and Sarah Ackerman for generously providing transgenic fly lines. We also thank Lauren Carlisle and Elaina Hildner for their invaluable technical support. This study is based upon work supported by the Texas A&M University Division of Research Targeted Proposal Teams (TPT) funding program, Zarin Ascend FY25 and FY26, as well as two internal grants from Texas A&M College of Arts and Sciences, Zarin STRP FY24 and FY25.Figure preparation: images in figures were prepared as Orthogonal Projections using Zen Blue software and assembled using Adobe Illustrator and Adobe Photoshop.

## Author contributions

Conceptualization, L.O. and A.Z.; data curation, L.O., P.M.M., K.K., S.A., A.S., and A.Z.; formal analysis, L.O. and A.Z.; funding acquisition, A.Z.; investigation, L.O. and A.Z.; methodology, L.O., P.M.M., K.K., S.A., A.S., and A.Z. project administration, L.O. and A.Z.; resources, L.O., P.M.M., K.K., S.A., A.S., and A.Z.; software, L.O., K.K., A.S., and A.Z.; supervision, A.Z.; validation, L.O., P.M.M., K.K., S.A., A.S., and A.Z.; visualization, L.O., P.M.M., K.K., S.A., A.S., and A.Z.; writing, original draft, L.O. and A.Z.; writing, review & editing, L.O. and A.Z.

## Declaration of interests

The authors declare no competing interests.

## Declaration of generative AI and AI-assisted technologies in the writing process

AI-assisted technologies were used exclusively for (1) grammar and language editing, and (2) performing sanity checks on MATLAB code developed by the authors. All original manuscript text was written and prepared by the authors prior to any interaction with AI tools. For grammar checks, only author-generated text was provided as input to AI-assisted technologies, and no text generated solely by AI was included in the manuscript. To verify MATLAB code functionality and ensure computational sanity, two independent AI tools (ChatGPT and Google Gemini) were used in parallel, strictly for code review, debugging suggestions, and logical validation. No content in this manuscript—including text, figures, results, or analyses—was generated *de novo* by AI. AI tools were used only to check, validate, and assist with human-generated content.

## STAR★Methods

### Key resources table

**Table:** 

REAGENT or RESOURCE	SOURCE	IDENTIFIER
**Antibodies**		

Rabbit polyclonal anti-GFP	Invitrogen	Catalog #A-11122; RRID: AB_221569
Mouse monoclonal anti-Dlg	DSHB	RRID: AB_528203
Rabbit polyclonal anti mCherry/tdTomato	Novus	Catalog# NBP2-25157; RRID:AB_2753204
Mouse monoclonal anti-GFP	DSHB	RRID: AB_2617418
Alexa Fluor 488 AffiniPure Goat polyclonal Anti-Hrp	Jackson ImmunoResearch	Code: 123-545-021; RRID: AB_2338965
Alexa Fluor 488 Goat polyclonal anti-Rabbit	Invitrogen	Catalog # A-11034; RRID: AB_2576217
Alexa Fluor 555 Goat polyclonal anti-Mouse	Invitrogen	Catalog # A32727; RRID: AB_2633276
Alexa Fluor 555 Goat polyclonal anti-Rabbit	Invitrogen	Catalog # A-21428; RRID: AB_2535849
Alexa Fluor 488 Goat polyclonal anti-Mouse	Invitrogen	Catalog # A-11001; RRID: AB_2534069
Alexa Fluor 405 Goat polyclonal anti-Mouse	Invitrogen	Catalog # A-31553; RRID: AB_221604

**Chemicals, peptides, and recombinant proteins**		

Phalloidin CF647	Biotium	Catalog # 00048

**Experimental models: Organisms/strains**		

D. melanogaster: w;94G06-Gal4	Bloomington^51^	BDSC #40701
D. melanogaster: w;94G06-LexA	Troy Littleton^43^^,^^51^	N/A
D. melanogaster: w;94G06:GFP	This paper	N/A
D. melanogaster: w; CQ-LexA	Cooney et al.^44^; Sweeney et al^54^	N/A
D. melanogaster: w; CQ-LexA; 94G06-Gal4	This paper	N/A
D. melanogaster: w; CQ-Gal4	Bloomington	BDSC #7468
D. melanogaster: w;27E09-Gal4	This paper	N/A
D. melanogaster: w; 27E09-LexA	This paper	N/A
D. melanogaster: w; 27E09-LexA; 94G06-Gal4	This paper	N/A
D. melanogaster: w; CQ-Gal4, 27E09-Gal4	This paper	N/A
D. melanogaster: w; CQ-Gal4, 27E09-Gal4; 94G06-LexA	This paper	N/A
D. melanogaster: w; CQ-Gal4; 94G06-Gal4	This paper	N/A
D. melanogaster: w; CQ-LexA, 27E09-LexA	This paper	N/A
D. melanogaster: w; CQ-LexA, 27E09-LexA; 94G06-LexA	This paper	N/A
D. melanogaster: w; CQ-LexA, 27E09-LexA; 94G06-Gal4	This paper	N/A
D. melanogaster: w;27E09-GFP	This paper	N/A
D. melanogaster: w;13xlexAop2rpr.H}attP5/CyO; Dr/TM6C, Sb	Robin Harris	BDSC #605696
D. melanogaster: w; LexAop-GtACR1.EGFP	Bloomington	BDSC #92985 or 605692
D. melanogaster: w; UAS-GTACR1-GFP	Bloomington	BDSC #: 92983
D. melanogaster: w; UAS-TNT.G2	Bloomington	BDSC #28838
D. melanogaster: w; UAS- BoNT-C	Dion Dickman^22^	N/A
D. melanogaster: w; LexAop-Kir2.1		N/A
D. melanogaster: w; UAS-RPR.hid, Tub-Gal80ts	Sarah Ackerman	N/A
D. melanogaster: w; UAS-RPR.hid, Tub-Gal80ts; 27E09-GFP	This paper	N/A
D. melanogaster: 10×UAS-myr:GFP	Bloomington	BDSC #32198
D. melanogaster: w; LexAop-myrGFP	Bloomington	BDSC #32212 BDSC #32209
D. melanogaster: w; LexAop-TdTomato	N/A	BDSC # 77139
D. melanogaster: w;44H10:GCamp6f	Huang and Zarin^45^	N/A
D. melanogaster: w; UAS-RPR.hid,Tub-Gal80ts; 44H10:GCamp6f	This Paper	N/A
D. melanogaster: w; 44H10:GCaMPf,UAS-GtACR1	Huang and Zarin^45^	N/A
D. melanogaster: w; lexAop2rpr.H}attP5; 44H10:GCaMPf,UAS-GtACR1	This Paper	N/A
D. melanogaster: w; lexAop2rpr.H}attP5/CyO; 44H10:GCamp6f/TM6C, Sb	This Paper	N/A
D. melanogaster: w;13xlexAop2rpr.H}attP5/CyO; UAS-myrGFP	This Paper	N/A
D. melanogaster: w; LexAop-GtACR1.EGFP/CyO; 44H10:GCamp6f/TM6C, Sb	This Paper	N/A
D. melanogaster: w; CQ-Gal4,27E09-Gal4; 94G06-lexA, LexAop-TdTomato	This Paper	N/A
D. melanogaster: w; CQ-Gal4, 27E09-Gal4;44H10:GCamp6f	This Paper	N/A
D. melanogaster: w; 20XUAS-6XmCherry HA}VK00018/CyO; Dr/TM6C, Sb,Tb	Bloomington	BDSC# 52267
D. melanogaster: w; SP/CYO; 13XLexAop2-6XmCherry-HA}attP2	Bloomington	BDSC #52271
D. melanogaster: w; lexAop2rpr.H}attP5/CYO; 13XLexAop2-6XmCherry-HA}attP2	This Paper	N/A

**Deposited data**		
Mendeley Data	This Paper	Mendeley Data: https://data.mendeley.com/datasets/c9fdgs69xx/2

**Software and algorithms**		

Fiji	N/A	https://imagej.net/software/fiji/downloads
Zeiss Zen Blue	N/A	https://www.zeiss.com/microscopy/us/products/software/zeiss-zen.html
Zeiss Zen lite	N/A	https://www.zeiss.com/microscopy/us/products/software/zeiss-zen-lite.html
Rstudio	N/A	https://rstudio-education.github.io/hopr/starting.html
NMJ Bouton Morphometrics	Schneck lab^41^	https://figshare.com/articles/dataset/Drosophila_NMJ_Morphometrics/2077399/1

**Other**		

Zeiss LSM900 Confocal Microscope	N/A	N/A
Zeiss Stemi 305 Microscope	N/A	N/A

### Experimental model and study participant details

#### Fly husbandry

All Crosses were reared at 25°C at 50% humidity with a 12 h light/dark cycle. For crosses with GTACR, larvae were fed with all-trans retinol (ATR) and kept in constant darkness until they were ready to be imaged or dissected. For crosses with GTACR, larvae not fed with ATR were used as one of the control groups.

Crosses involving RPR were at 29°C to ensure ablation efficiency. For Larval ablation using Gal80ts, Embryos were kept at 25°C and as larvae hatched, they transferred to 29°C to induce ablation. A list of stocks used can be found in key resources table. Due to a significant developmental delay in larvae with motor neuron ablation, developmental stage was determined from the larvae size and not time.

### Method details

#### Fillet dissection

In HL3.1 (hemolymph-like solution), late 3^rd^ instar larvae were pinned at the head and the tail with the ventral side up. Spring scissors were used to cut the larvae up the ventral midline and the body wall was cleaned. Four additional pins were used to splay the body wall open and the HL3.1 was removed.

#### Immunohistochemistry

Fillet dissections were fixed with 4% paraformaldehyde (PFA) for 10 min. After washing with PBST, samples were blocked using equal parts 5% Goat serum and 5% Donkey serum in PBST overnight at 4°C. Samples were incubated with primary antibodies (see Table S2) at room temperature for 2hrs or overnight at 4°C. After washing, secondary antibodies (see Table S2) were incubated at room temperature for 2hrs or overnight at 4°C. Dissections were washed in PBST, mounted in flouromont-G on microscope slides and stores at −20°C until imaging.

#### NMJ imaging

Using a Zeiss LSM900 Confocal microscope equipped with a 40× objective, Airyscan images were acquired in super resolution (SR) with the Zen Blue software. Raw Z-stacks were processed in Zen blue with 3D Airyscan Processing with automatic filter. Two hemisegments (A1-A7) were imaged from each animal and 6–8 animals from each condition. The example z stack of an entire animal was acquired using the 5× objective with light sheet microscopy. For the Gal80ts data in Figure 5, images were randomized and blinded for genotype before being analyzed to mitigate bias.

#### Peristalsis assay

Third-instar larvae crawled along grooves on a 2% agarose plate placed over a graph sheet with start and end markers spaced 1.00 cm apart. Videos were acquired from directly overhead under constant illumination at room temperature. See *Situala* et al.^53^ for more details on the illumination device and iPhone camera setup and installation on the dissection scope. All groups were recorded and analyzed under identical temperature and illumination; camera height and view angle were fixed to minimize parallax. Trials with mid-lane stops, reversals, collisions, or loss of focus were excluded *a priori*. Files with variable frame rate were converted to a constant frame rate prior to analysis when needed.

#### Intact larvae imaging

To track branching across the larval stages, L0, L1, L2 and L3 larvae were washed in distilled water. Larvae were immobilized by a coverslip pressing them into a 2% agarose gel pad on a microscope slide. Depending on the size of the larvae, the 20×, 10×, or 5× objective was used to acquire a z stack of the body wall to visualize the marked axon. Every abdominal axon from 5 animals at each stage were imaged and branching was quantified as described in NMJ quantification for each condition except branch points were not separated between axon and NMJ. Muscle calcium imaging was conducted with L3 larvae as described above except with a 5× objective acquiring a time series as larvae complete peristalsis.

### Quantification and statistical analysis

#### Phenotype quantification

Abdominal segments 1–7 (A1-7) were examined in stained animals, any animal with fewer than 6 intact segments were disregarded. Muscle innervation was determined by the colocalization of the marked axon and dlg on different muscles. Any segment with ectopic innervation was considered Branched and any segment where the muscles were intact and the axon and Dlg were missing were considered Missing. For each animal the percentage of segments with a phenotype or innervating a muscle were divided by the total number of intact muscles and multiplied by 100 to get the percentage of sections. The number of DL muscles innervated was counted for each section.

#### NMJ quantification

*Bouton Morphology.* In Fiji, a manual threshold was set to select the axon of the GFP channel, that selection was used as a mask on the Dlg channel to identify the post synapses belonging to the marked axon. All Dlg not indicated by the mask were cleared before running analysis. The Drosophila NMJ Morphometrics Fiji plugin developed by the Schneck lab^40^^,^^41^ was used to quantify the area. The macro completed background subtraction with a rolling ball radius set to 200. NMJ outline for area was determined via built in thresholding methods, Reny Entropy was most commonly used but if the resulting outline was incorrect different methods were tested until the outline was tight around the boutons. ROIs were used to separately analyze the post synapses on each muscle. Using the dlg and GFP channels and a composite of the two, individual boutons were counted based on identification of distinct circles as exemplified in Figure S1. The area from all 4 muscles were added to get the total of each hemisegment. The average bouton size for each hemisegment was determined by dividing the total area by the total bouton number.

*Branching Points*. Segments of axon colocalized with Dlg and independent of DLG were considered separately. The number of branching points were counted and categorized based on their degree of separation from the main stalk, with direct branches being 1^st^ order, branches off a 1^st^ order branch being 2^nd^ order, and branches off a 2^nd^ order branch being 3^rd^ order. All 1^st^, 2^nd^, and 3^rd^ order branches were added to get the total number of axon or NMJ branch points.

#### Peristalsis quantification

*Wave definition.* A forward peristaltic wave was defined as the onset of an posterior constriction that propagates tailward and completes at the anterior end without reversal or pause. Only forward waves were scored.

*Peristalsis efficiency (cm per wave).* The traverse used for scoring began when the larval head touched the start line and ended when it crossed the end line (1.00 cm). Using frame stepping (Option + Right/Left Arrow) to navigate precisely, the number of forward waves (N) required to traverse 1.00 cm was counted. Peristalsis efficiency was computed as:

$$\text{efficiency}=\frac{1.00\;\text{cm}}{N}\left(\text{cm}/\text{wave}\right)$$

*Peristalsis duration (s per wave).* For each wave, boundaries were set with Edit → Trim … so the window contained only that wave (start at posterior constriction onset; end at tail completion). The Duration field in Movie Inspector was recorded in seconds (millisecond precision). As an equivalent frame-based check, duration can be computed as Δframes/FPS after stepping frame-by-frame; frame counts are integers.

#### Muscle calcium imaging quantification

*Gcamp signal intensity*. A custom MATLAB script^39^ was used where an ROI was placed on each muscle and placement was adjusted for each frame, avoiding overlapping sections. The calcium signal (ΔF/F) for each muscle was calculated using the formula (F - F_0_)/F_0_, where F is the fluorescence at a given time, and F_0_ is the baseline, defined as the 10th percentile of values from the first third of the recording.

*Muscle contraction*. A custom MATLAB script^39^ was used where a line ROI was placed with the ends at the segment boundaries and the placement of the endpoints was adjusted for each frame. The normalized change in muscle length was calculated by subtracting the maximum length by the minimum length and dividing by the maximum.

### Statistical analysis

Data analysis was performed in MATLAB and R. For experiments that involved either repeated sampling of multiple hemisegments per larva or longitudinal tracking of the same hemisegments from L1 to wandering L3 (Figure 1, Figure 5), linear mixed-effects (LME) models were used with each individual larva as a random intercept. Fixed effects were specified per design and included Stage, Genotype, and Stage×Genotype interactions, or Genotype alone for single-factor contrasts. Matched L1→wandering L3 contrasts and first-versus second-order branch comparisons within each stage were implemented in MATLAB using paired, two-group t-tests (default two-tailed). Normality was assessed using Shapiro–Wilk. Multi-group comparisons of NMJ morphometry, axon branchpoint number, and calcium imaging or locomotion parameters were performed using Kruskal Wallis with Dunn post-hoc test for non-parametric data and Oneway ANOVA with Tukey HSD post-hoc for parametric data when a panel included more than two genotypes. For unpaired two-group comparisons, statistical tests were selected based on data distribution: Student’s *t* test (two-tailed) was used for normally distributed data, and the Wilcoxon rank-sum test was used for non-normally distributed data. The test used for each graph is indicated in the figure legend. Summary data are reported as mean ± SEM, with replicates provided in figure legends. Statistical significance thresholds were defined as *p* < 0.05 (∗*), <0.01 (*∗∗*), <0.001 (*∗∗∗), or <0.0001 (∗∗∗∗).

## Supplemental information

Video S7. Silencing the sprouted MN1 abolishes reinnervated muscle activity In larvae expressing GtACR1 in MN1 after CQUIs-lexA > Aop-RPR-mediated ablation (CQ ∪ Is-lexA > Aop-RPR; MN1-Gal4 > UAS-GtACR1), light activation of GtACR1 eliminates all DL muscle activity. This confirms that reinnervation and functional output in previously denervated muscles depend on the activity of the sprouted MN1 terminals.

Video S8. Peristalsis efficiency assay comparing different genotypes Side-by-side recordings of larval crawling behavior under different experimental conditions illustrate the impact of MN ablation, silencing, and MN1 reinnervation on locomotor efficiency. Genotypes (left to right): 1. MN1 ∪ CQ ∪ Is-lexA > Aop-GtACR1, ATR– (control). 2. MN1 ∪ CQ ∪ Is-lexA > Aop-RPR (full ablation of all DL-innervating MNs). 3. MN1 ∪ CQ ∪ Is-lexA > Aop-GtACR1, ATR+ (all DL MNs acutely silenced). 4. CQ ∪ Is-lexA > Aop-RPR (sprouted MN1 present). 5. CQ ∪ Is-lexA > Aop-GtACR1, ATR+ (MN1 intact but neighboring MNs silenced). 6. CQ ∪ Is-lexA > Aop-RPR; MN1-Gal4 > UAS-GtACR1, ATR+ (sprouted MN1 acutely silenced). This assay visually demonstrates the functional consequences of MN ablation, silencing, and reinnervation on coordinated locomotor output.

## References

[1] Edds M.V. (1949). Experiments on partially deneurotized nerves; hypertrophy of residual fibers. J. Exp. Zool..

[2] Edds M.V. (1953). Collateral nerve regeneration. Q. Rev. Biol..

[3] Slack J.R., Hopkins W.G., Williams M.N. (1979). Nerve sheaths and motoneurone collateral sprouting. Nature.

[4] Bowling D., Nicholls J., Parnas I. (1978). Destruction of a single cell in the central nervous system of the leech as a means of analysing its connexions and functional role. J. Physiol..

[5] Parnas I. (1987). Strengthening of synaptic inputs after elimination of a single neurone innervating the same target. J. Exp. Biol..

[6] Dudel J., Parnas I. (1987). Augmented synaptic release by one excitatory axon in regions in which a synergistic axon was removed in lobster muscle. J. Physiol..

[7] Parnas I., Dudel J., Cohen I., Franke C. (1984). Strengthening of synaptic contacts of an excitatory axon on elimination of a second excitatory axon innervating the same target. J. Neurosci..

[8] Büschges A., Djokaj S., Bässler D., Bässler U., Rathmayer W. (2000). Neuromuscular plasticity in the locust after permanent removal of an excitatory motoneuron of the extensor tibiae muscle. J. Neurobiol..

[9] Frank C.A., Wang X., Collins C.A., Rodal A.A., Yuan Q., Verstreken P., Dickman D.K. (2013). New approaches for studying synaptic development, function, and plasticity using Drosophila as a model system. J. Neurosci..

[10] Menon K.P., Carrillo R.A., Zinn K. (2013). Development and plasticity of the Drosophila larval neuromuscular junction. Wiley interdisciplinary reviews. Developmental biology.

[11] Davis G.W., Müller M. (2015). Homeostatic control of presynaptic neurotransmitter release. Annu. Rev. Physiol..

[12] Harris K.P., Littleton J.T. (2015). Transmission, Development, and Plasticity of Synapses. Genetics.

[13] Newman Z.L., Hoagland A., Aghi K., Worden K., Levy S.L., Son J.H., Lee L.P., Isacoff E.Y. (2017). Input-Specific Plasticity and Homeostasis at the Drosophila Larval Neuromuscular Junction. Neuron.

[14] Genç Ö., Davis G.W. (2019). Target-wide Induction and Synapse Type-Specific Robustness of Presynaptic Homeostasis. Curr. Biol..

[15] Goel P., Dufour Bergeron D., Böhme M.A., Nunnelly L., Lehmann M., Buser C., Walter A.M., Sigrist S.J., Dickman D. (2019). Homeostatic scaling of active zone scaffolds maintains global synaptic strength. J. Cell Biol..

[16] James T.D., Zwiefelhofer D.J., Frank C.A. (2019). Maintenance of homeostatic plasticity at the Drosophila neuromuscular synapse requires continuous IP(3)-directed signaling. eLife.

[17] Frank C.A., James T.D., Müller M. (2020). Homeostatic control of Drosophila neuromuscular junction function. Synapse.

[18] Wang Y., Lobb-Rabe M., Ashley J., Anand V., Carrillo R.A. (2021). Structural and Functional Synaptic Plasticity Induced by Convergent Synapse Loss in the Drosophila Neuromuscular Circuit. J. Neurosci..

[19] Li X., Chien C., Han Y., Sun Z., Chen X., Dickman D. (2021). Autocrine inhibition by a glutamate-gated chloride channel mediates presynaptic homeostatic depression. Sci. Adv..

[20] Yeates C.J., Frank C.A. (2021). Homeostatic Depression Shows Heightened Sensitivity to Synaptic Calcium. Front. Cell. Neurosci..

[21] Armstrong N.S., Frank C.A. (2022). The calcineurin regulator Sarah enables distinct forms of homeostatic plasticity at the Drosophila neuromuscular junction. Front. Synaptic Neurosci..

[22] Han Y., Chien C., Goel P., He K., Pinales C., Buser C., Dickman D. (2022). Botulinum neurotoxin accurately separates tonic vs. phasic transmission and reveals heterosynaptic plasticity rules in Drosophila. eLife.

[23] He K., Han Y., Li X., Hernandez R.X., Riboul D.V., Feghhi T., Justs K.A., Mahneva O., Perry S., Macleod G.T., Dickman D. (2023). Physiologic and Nanoscale Distinctions Define Glutamatergic Synapses in Tonic vs Phasic Neurons. J. Neurosci..

[24] Wang Y., Zhang R., Huang S., Valverde P.T.T., Lobb-Rabe M., Ashley J., Venkatasubramanian L., Carrillo R.A. (2023). Glial Draper signaling triggers cross-neuron plasticity in bystander neurons after neuronal cell death in Drosophila. Nat. Commun..

[25] Wang T., Frank C.A. (2025). Using Electrophysiology to Study Homeostatic Plasticity at the Drosophila Neuromuscular Junction. Cold Spring Harb. Protoc..

[26] Aponte-Santiago N.A., Ormerod K.G., Akbergenova Y., Littleton J.T. (2020). Synaptic Plasticity Induced by Differential Manipulation of Tonic and Phasic Motoneurons in Drosophila. J. Neurosci..

[27] Yifu Han C.C., Goel P., He K., Pinales C., Buser C., Dickman D. (2022). Botulinum neurotoxin accurately separates tonic vs phasic transmission and reveals heterosynaptic plasticity rules in Drosophila. eLife.

[28] Lu Z., Chouhan A.K., Borycz J.A., Lu Z., Rossano A.J., Brain K.L., Zhou Y., Meinertzhagen I.A., Macleod G.T. (2016). High-Probability Neurotransmitter Release Sites Represent an Energy-Efficient Design. Curr. Biol..

[29] Lnenicka G.A., Keshishian H., New Collective Author (2000). Identified motor terminals in Drosophila larvae show distinct differences in morphology and physiology. J. Neurobiol..

[30] Aponte-Santiago N.A., Littleton J.T. (2020). Synaptic Properties and Plasticity Mechanisms of Invertebrate Tonic and Phasic Neurons. Front. Physiol..

[31] Dum R.P., Kennedy T.T. (1980). Physiological and histochemical characteristics of motor units in cat tibialis anterior and extensor digitorum longus muscles. J. Neurophysiol..

[32] Atwood H. (2008). Parallel ‘phasic' and ‘tonic' motor systems of the crayfish abdomen. J. Exp. Biol..

[33] Landgraf M., Bossing T., Technau G.M., Bate M. (1997). The origin, location, and projections of the embryonic abdominal motorneurons of Drosophila. J. Neurosci..

[34] Hoang B., Chiba A. (2001). Single-cell analysis of Drosophila larval neuromuscular synapses. Dev. Biol..

[35] Landgraf M., Jeffrey V., Fujioka M., Jaynes J.B., Bate M. (2003). Embryonic origins of a motor system: motor dendrites form a myotopic map in Drosophila. PLoS Biol..

[36] Landgraf M., Thor S. (2006). Development of Drosophila motoneurons: specification and morphology. Semin. Cell Dev. Biol..

[37] Zwart M.F., Pulver S.R., Truman J.W., Fushiki A., Fetter R.D., Cardona A., Landgraf M. (2016). Selective Inhibition Mediates the Sequential Recruitment of Motor Pools. Neuron.

[38] Zarin A.A., Labrador J.P. (2017). Motor axon guidance in Drosophila. Semin. Cell Dev. Biol..

[39] Zarin A.A., Mark B., Cardona A., Litwin-Kumar A., Doe C.Q. (2019). A multilayer circuit architecture for the generation of distinct locomotor behaviors in Drosophila. eLife.

[40] Nijhof B., Castells-Nobau A., Wolf L., Scheffer-de Gooyert J.M., Monedero I., Torroja L., Coromina L., van der Laak J.A.W.M., Schenck A. (2016). A New Fiji-Based Algorithm That Systematically Quantifies Nine Synaptic Parameters Provides Insights into Drosophila NMJ Morphometry. PLoS Comput. Biol..

[41] Castells-Nobau A., Nijhof B., Eidhof I., Wolf L., Scheffer-de Gooyert J.M., Monedero I., Torroja L., van der Laak J., Schenck A. (2017). Two Algorithms for High-throughput and Multi-parametric Quantification of Drosophila Neuromuscular Junction Morphology. J. Vis. Exp..

[42] Jetti S.K., Crane A.B., Akbergenova Y., Aponte-Santiago N.A., Cunningham K.L., Whittaker C.A., Littleton J.T. (2023). Molecular logic of synaptic diversity between Drosophila tonic and phasic motoneurons. Neuron.

[43] Heckscher E.S., Zarin A.A., Faumont S., Clark M.Q., Manning L., Fushiki A., Schneider-Mizell C.M., Fetter R.D., Truman J.W., Zwart M.F. (2015). Even-Skipped(+) Interneurons Are Core Components of a Sensorimotor Circuit that Maintains Left-Right Symmetric Muscle Contraction Amplitude. Neuron.

[44] Cooney P.C., Huang Y., Li W., Perera D.M., Hormigo R., Tabachnik T., Godage I.S., Hillman E.M.C., Grueber W.B., Zarin A.A. (2023). Neuromuscular basis of Drosophila larval rolling escape behavior. Proc. Natl. Acad. Sci. USA.

[45] Huang Y., Zarin A.A. (2022). A premotor microcircuit to generate behavior-specific muscle activation patterns in Drosophila larvae. bioRxiv.

[46] Goyal L., McCall K., Agapite J., Hartwieg E., Steller H. (2000). Induction of apoptosis by Drosophila reaper, hid and grim through inhibition of IAP function. EMBO J..

[47] McGuire S.E., Le P.T., Osborn A.J., Matsumoto K., Davis R.L. (2003). Spatiotemporal rescue of memory dysfunction in Drosophila. Science (New York, N.Y.).

[48] Suster M.L., Seugnet L., Bate M., Sokolowski M.B. (2004). Refining GAL4-driven transgene expression in Drosophila with a GAL80 enhancer-trap. Genesis.

[49] Carreira-Rosario A., Zarin A.A., Clark M.Q., Manning L., Fetter R.D., Cardona A., Doe C.Q. (2018). MDN brain descending neurons coordinately activate backward and inhibit forward locomotion. eLife.

[50] Pérez-Moreno J.J., O'Kane C.J. (2019). GAL4 Drivers Specific for Type Ib and Type Is Motor Neurons in Drosophila. G3 (Bethesda, Md.).

[51] Mauss A.S., Busch C., Borst A. (2017). Optogenetic Neuronal Silencing in Drosophila during Visual Processing. Sci. Rep..

[52] Mohammad F., Stewart J.C., Ott S., Chlebikova K., Chua J.Y., Koh T.W., Ho J., Claridge-Chang A. (2017). Optogenetic inhibition of behavior with anion channelrhodopsins. Nat. Methods.

[53] Sitaula A., Huang Y., Zarin A. (2024). Application of a Dual Optogenetic Silencing-Activation Protocol to Map Motor Neurons Driving Rolling Escape Behavior in Drosophila Larvae. Bio. Protoc..

[54] Sweeney S.T., Broadie K., Keane J., Niemann H., O'Kane C.J. (1995). Targeted expression of tetanus toxin light chain in Drosophila specifically eliminates synaptic transmission and causes behavioral defects. Neuron.

[55] Baines R.A., Uhler J.P., Thompson A., Sweeney S.T., Bate M. (2001). Altered electrical properties in Drosophila neurons developing without synaptic transmission. J. Neurosci..

[56] Johns D.C., Marx R., Mains R.E., O’Rourke B., Marbán E. (1999). Inducible Genetic Suppression of Neuronal Excitability. J. Neurosci..

[57] Nadeau H., McKinney S., Anderson D.J., Lester H.A. (2000). ROMK1 (Kir1.1) causes apoptosis and chronic silencing of hippocampal neurons. J. Neurophysiol..

[58] Brown M.C., Holland R.L. (1979). A central role for denervated tissues in causing nerve sprouting. Nature.

[59] Chang T.N., Keshishian H., New Collective Author (1996). Laser ablation of Drosophila embryonic motoneurons causes ectopic innervation of target muscle fibers. J. Neurosci..

[60] Bate M. (1990). The embryonic development of larval muscles in Drosophila. Development.

[61] Pal S., Hartnett K.A., Nerbonne J.M., Levitan E.S., Aizenman E. (2003). Mediation of neuronal apoptosis by Kv2.1-encoded potassium channels. J. Neurosci..

[62] Repnikova E., Koles K., Nakamura M., Pitts J., Li H., Ambavane A., Zoran M.J., Panin V.M. (2010). Sialyltransferase regulates nervous system function in Drosophila. J. Neurosci..

[63] Giachello C.N.G., Hunter I., Pettini T., Coulson B., Knüfer A., Cachero S., Winding M., Arzan Zarin A., Kohsaka H., Fan Y.N. (2022). Electrophysiological validation of monosynaptic connectivity between premotor interneurons and the aCC motoneuron in the Drosophila larval CNS. J. Neurosci..

[64] Modney B.K., Muller K.J. (1994). Novel synapses compensate for a neuron ablated in embryos. Proc. Biol. Sci..

[65] Hilton B.J., Griffin J.M., Fawcett J.W., Bradke F. (2024). Neuronal maturation and axon regeneration: unfixing circuitry to enable repair. Nat. Rev. Neurosci..

