# Supplementary material for: Complete muscle denervation triggers compensatory sprouting of a bystander tonic motor neuron in Drosophila larvae
Source: iScience. 2026 Jul 27;29(8):116099. doi: 10.1016/j.isci.2026.116099 (PMC13446351; doi:10.1016/j.isci.2026.116099)
Supplement: Supplementary file 1 [file mmc1.pdf]

iScience, Volume *29*

Supplemental information

Complete muscle denervation triggers compensatory sprouting of a bystander tonic motor neuron in *Drosophila* larvae

Lizzy Olsen, Parinita Mitchelle Mandhyan, Komal Kaur, Sara Arredondo, Ankura Sitaula, and Aref Zarin

This PDF file includes:

Figures S1 to S3
Legends for Movies S1 to S8
SI References

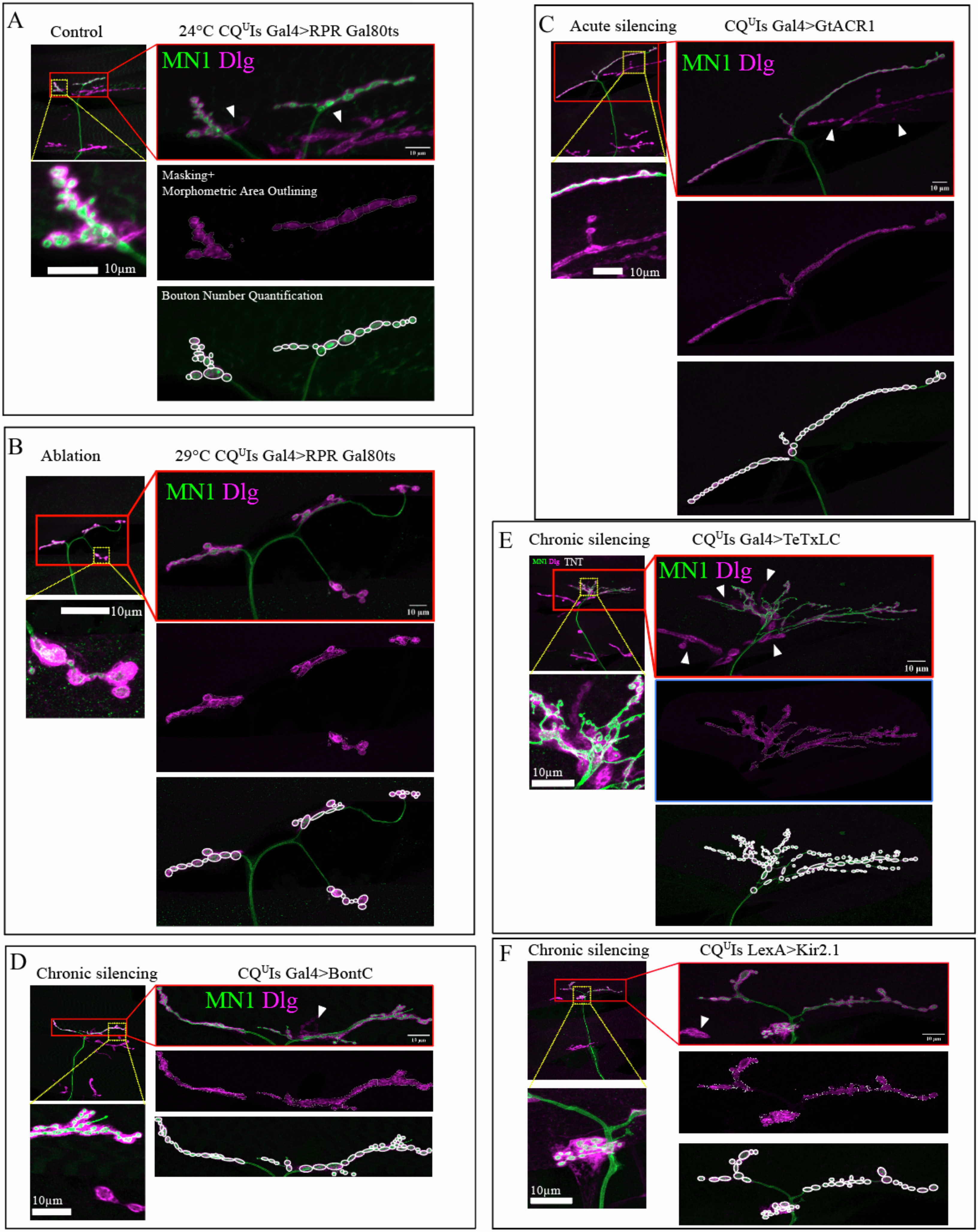

A
Control
24°C CQ^U Is Gal4>RPR Gal80ts
MN1 Dlg
10μm
Masking+
Morphometric Area Outlining
Bouton Number Quantification
B
Ablation
29°C CQ^U Is Gal4>RPR Gal80ts
MN1 Dlg
10μm
C
Acute silencing
CQ^U Is Gal4>GtACR1
MN1 Dlg
10μm
D
Chronic silencing
CQ^U Is Gal4>BontC
MN1 Dlg
10μm
E
Chronic silencing
CQ^U Is Gal4>TeTxLC
MN1 Dlg TNT
MN1 Dlg
10μm
F
Chronic silencing
CQ^U Is LexA>Kir2.1
10μm

Fig. S1 (related to main Fig 3). Workflow illustrating the NMJ analysis pipeline [adapted from [1, 2]] in different larval groups. Morphological responses of MN1 to distinct manipulations of neighboring motor neurons.

Representative images illustrate the structural remodeling of MN1 following ablation or silencing of adjacent tonic and phasic motor neurons (MNs), as visualized with membrane-targeted GFP and Dlg immunostaining.

(A) In temperature-controlled **unmanipulated controls** (CQ ∪ Is-Gal4 > UAS-RPR, Gal80ts; raised entirely at 24°C), MN1 displays its typical morphology, forming a compact NMJ exclusively on its native target, Muscle 1. No ectopic projections or abnormal branching are detected.

(B) In the **ablation group** (CQ ∪ Is-Gal4 > UAS-RPR, Gal80ts; raised at 29°C), selective apoptosis of neighboring MNs induces a dramatic response in MN1. The axon extends beyond its native territory and establishes new, ectopic NMJs on denervated Muscles 2, 9, and 10, demonstrating robust sprouting and target reinnervation.

(C) In the **acute silencing condition** (CQ ∪ Is-Gal4 > UAS-GtACR1; light-exposed), MN1 remains restricted to Muscle 1. Although NMJ area and bouton number are elevated relative to controls, MN1 does not form branches onto adjacent muscles.

(D) In the **chronic silencing group targeting both evoked and spontaneous release** (CQ ∪ Is-Gal4 > UAS-BoNT-C), MN1 similarly exhibits increased bouton numbers but no ectopic sprouting. The NMJ remains confined to its native muscle target, and axon branching remains limited.

(E) In the **chronic silencing group that blocks only evoked transmission** (CQ ∪ Is-Gal4 > UAS-TeTxLC), MN1 shows the most extensive remodeling without sprouting. NMJ area, bouton number, and terminal branching complexity are all significantly increased compared to controls and other silencing groups. However, bouton size is notably reduced. Importantly, the tonic and phasic MNs targeted by TeTxLC appear morphologically normal (white arrows), suggesting that structural changes in MN1 are not due to degeneration of neighboring neurons but rather reflect a plastic response to altered circuit activity.

(F) In the chronic silencing group that hyperpolarizes the membrane (CQ ∪ Is-LexA > LexAop-Kir), MN1 forms small ectopic synapses on muscles 2, 9, and 10, while increasing the area and bouton number.

Scale bars and genotype-specific labeling are provided in each panel. This figure complements the quantitative analysis shown in **main Figure 3**.

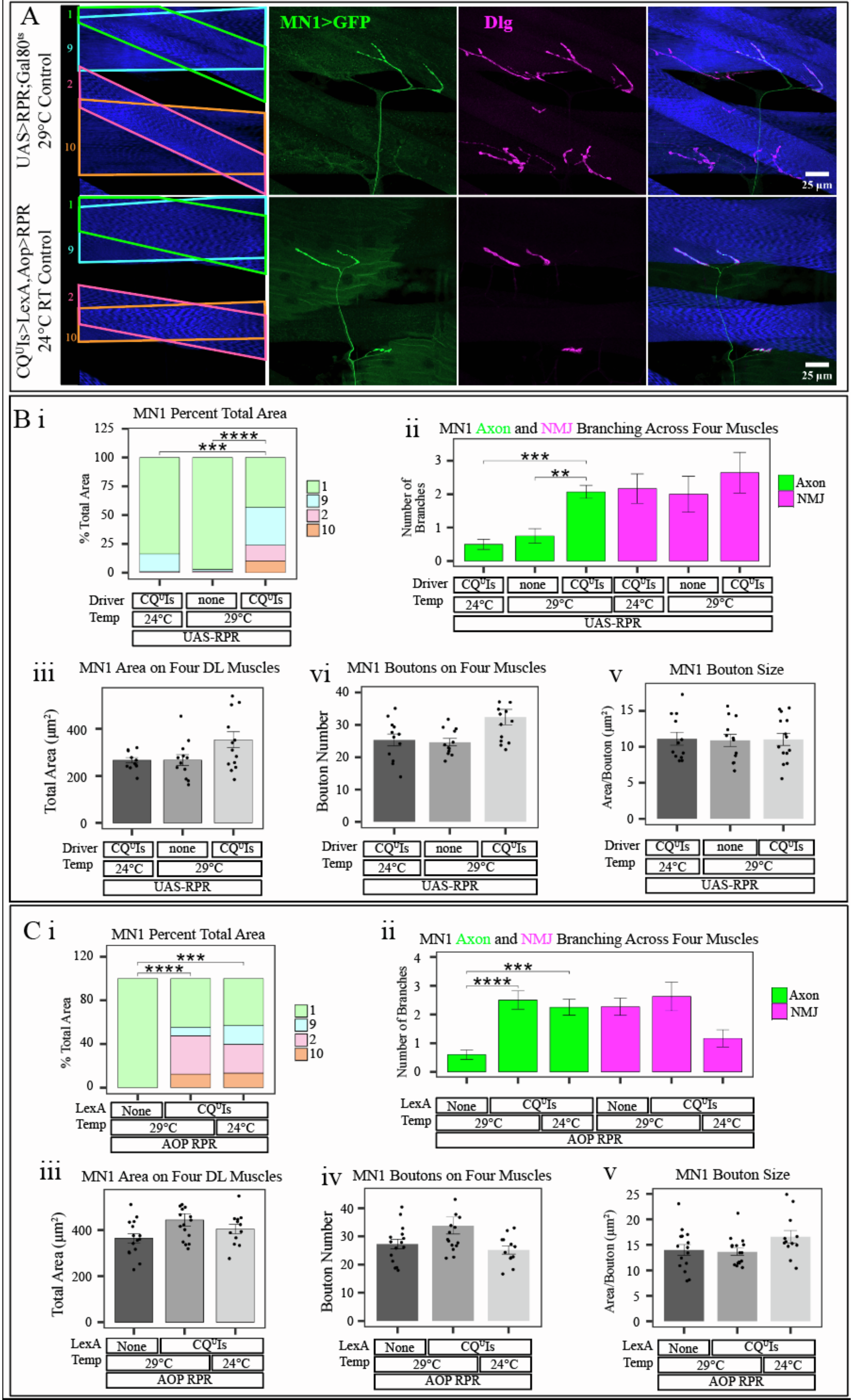
A
UAS>RPR;Gal80ts 29°C Control
CQUIs>LexA,Aop>RPR 24°C RT Control
MN1>GFP
Dlg
25 µm
B i
MN1 Percent Total Area
ii
MN1 Axon and NMJ Branching Across Four Muscles
iii
MN1 Area on Four DL Muscles
vi
MN1 Boutons on Four Muscles
V
MN1 Bouton Size
Driver
Temp
UAS-RPR
C i
MN1 Percent Total Area
ii
MN1 Axon and NMJ Branching Across Four Muscles
iii
MN1 Area on Four DL Muscles
iv
MN1 Boutons on Four Muscles
V
MN1 Bouton Size
LexA
Temp
AOP RPR
% Total Area
Number of Branches
Total Area (µm²)
Bouton Number
Area/Bouton (µm²)
Axon
NMJ

Fig. S2. (related to main Fig. 1, Fig. 3, and Fig. 5). Elevated temperature alone does not induce the MN1 sprouting phenotype.

(A) Representative immunostaining of MN1 axon morphology and NMJs.
Top: In the absence of a Gal4 driver, MN1 axons show no detectable sprouting or ectopic NMJ formation in control larvae (*UAS-Rpr/+*, reared continuously at 29 °C).
Bottom: Apoptotically denervated larvae (*CQ ∪ Is-LexA > LexAop-Rpr/+*, reared continuously at 24 °C) display robust MN1 heterosynaptic sprouting and ectopic NMJ formation on dorsal-longitudinal (DL) body wall muscles.

(B-C) Quantification of MN1 axon structure and NMJ morphometry across temperature and driver conditions.
(B i-v) Larvae carrying *UAS-Rpr* under *tub-Gal80^{ts}* regulation were analyzed in three groups:
(a) no Gal4 driver, continuously reared at 29 °C — no MN ablation occurs, and MN1 does not sprout and form ectopic NMJs on non-native target muscles 2, 9, and 10. Therefore, elevated temperature by itself does not induce the MN1 axon sprouting phenotype. The MN1 show an increase in NMJs branching on its native Muscle-1.
(b) *CQ ∪ Is*-Gal4 present, continuously reared at 24 °C — Gal80^{ts} suppresses Gal4 mediated expression of *UAS-Rpr*, blocking MN denervation and preventing MN1 sprouting. MN1 does not sprout and form ectopic NMJs on non-native target muscles 2, 9, and 10.
(c) *CQ ∪ Is*-Gal4 present, continuously reared at 29 °C — *Gal80^{ts}* becomes inactive. Gal4 activates UAS-RPR expression. MN ablation occurs, and the bystander MN1 form ectopic NMJs on muscles 2, 9, and 10 as well as its own native Muscle 1.

(C i-v) Larvae carrying *Aop-RPR* were analyzed in three groups:
(a) no *LexA* driver, continuously reared at 29 °C — no MN ablation occurs, and MN1 does not sprout and form ectopic NMJs on non-native target muscles 2, 9, and 10. Therefore, elevated temperature by itself does not induce the MN1 axon sprouting phenotype. The MN1 show an increase in NMJs branching on its native Muscle-1.
(b) *CQ ∪ Is- LexA* present, continuously reared at 24 °C — MN ablation occurs, and the bystander MN1 form ectopic NMJs on muscles 2, 9, and 10 as well as its own native Muscle 1.
(c) *CQ ∪ Is- LexA* present, continuously reared at 29 °C — *Aop-RPR* is expressed, MN ablation occurs, and the bystander MN1 form ectopic NMJs on muscles 2, 9, and 10 as well as its own native Muscle 1.
All Groups, n= 6 (12 hemisegments). Data points represent individual hemisegments. Error bars indicate mean ± SEM. Statistical analysis Biii-iv: One-way ANOVA test with Tukey HSD post-hoc test. Statistical analysis Bi-ii,v,C: Kuriskal Wallis with Dunn's post hoc test. (*$p<0.05$; **$p<0.01$; ***$p<0.001$; ****$p<0.0001$).

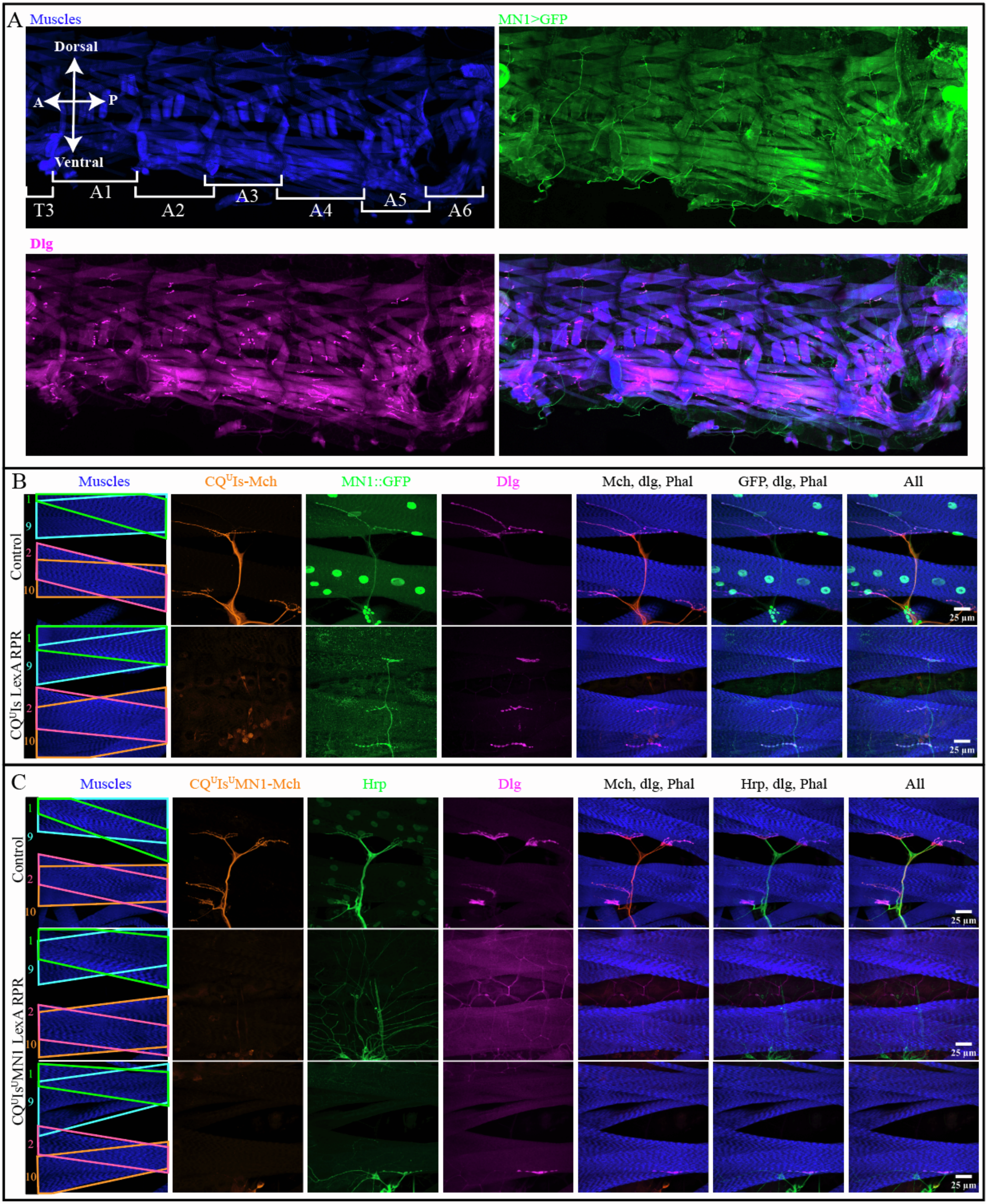

A
Muscles
MN1>GFP
Dorsal
Ventral
A
P
T3
A1
A2
A3
A4
A5
A6
Dlg
B
Muscles
CQUIs-Mch
MN1::GFP
Dlg
Mch, dlg, Phal
GFP, dlg, Phal
All
Control
CQUIs LexA RPR
1
9
2
10
25 µm
C
Muscles
CQUIsUMN1-Mch
Hrp
Dlg
Mch, dlg, Phal
Hrp, dlg, Phal
All
Control
CQUIsUMN1 LexA RPR
25 µm

Fig. S3. (related to main Fig. 1, Fig. 3, and Fig. 5). The axons of MNs expressing RPR are cleared in the periphery.

(A) A widefield view of six consecutive abdominal segments from a CQ ∪ Is > RPR larval fillet preparation stained for muscles (phalloidin, blue), NMJs (Dlg, magenta), and the bystander MN1 (MN1-Gal4 > UAS-myr-GFP). MN1 shows ectopic sprouting in segments A1–A3 and A5, while MN1 axons and Dlg are absent from the DL muscle field in segment A4, indicating a failure to reach both its native and ectopic dorsal targets. All remaining Dlg signal in DL muscles 1, 2, 9, and 10 co-localizes with MN1-GFP, and Dlg from ablated MN-Is and CQ MNs is no longer detectable, consistent with clearance of apoptotic MN axons in the periphery.

(B) Top row: mCherry expression under validated MN-LexA drivers (CQ ∪ Is-LexA > AOP-mCherry) labels motor axons in the expected muscle fields, confirming driver specificity and the absence of ectopic innervation when apoptosis is not induced. Bottom row: Co-expression of RPR and mCherry under the same drivers (CQ ∪ Is-LexA > AOP-RPR; AOP-mCherry) induces efficient ablation of the targeted MNs, producing mCherry-positive apoptotic debris in the periphery. Loss of Dlg signal at corresponding NMJs confirms that postsynaptic structures belonging to the ablated MNs are removed prior to MN1 quantification. MN1-GFP visualizes the bystander MN1 axon, which is not targeted for apoptosis. The bystander MN1-GFP maintains innervation of native muscle 1 while forming new NMJs on ectopic, denervated muscles 2, 9, and 10.

(C) Top row: mCherry driven by MN1 ∪ CQ ∪ Is-LexA (AOP-mCherry) labels motor projections in the expected dorsal longitudinal muscle fields without inducing apoptosis. Middle and Bottom row: mCherry and RPR co-expression under MN1 ∪ CQ ∪ Is-LexA (AOP-RPR; AOP-mCherry) ablates the intended population, producing mCherry-positive debris. Dlg signal is absent on DL muscles, indicating effective ablation and local clearance, with the exception of residual Dlg signals detected in a single muscle at the bottom panel. HRP immunostaining labels the entirety of the peripheral nervous system, including all motor axons, sensory dendrites, and non-targeted neurons, confirming that other projections remain structurally intact. Analyses were restricted to abdominal segments used for MN1 NMJ measurements.

Video S1. Control larva exhibits coordinated DL muscle activity during crawling. In an unperturbed animal (MN1 ∪ CQ ∪ Is-lexA > Aop-GtACR1, ATR–), GCaMP6f fluorescence reveals robust and sequential activation of dorsal longitudinal (DL) muscles. Calcium signals initiate in posterior segments and propagate anteriorly, reflecting normal peristaltic waves during forward locomotion.

Video S2. Complete ablation of DL-innervating MNs abolishes DL muscle activity. In larvae with all tonic and phasic DL-innervating MNs ablated (MN1 ∪ CQ ∪ Is-lexA > Aop-RPR), DL muscle contractions and GCaMP6f signals are completely absent, confirming effective denervation. Activity in ventral and lateral muscles remains intact, highlighting lexA-driver specificity.

Video S3. Acute silencing of all DL-innervating MNs eliminates DL muscle activity. Following light-dependent activation of GtACR1 in MN1, CQ, and Is neurons (MN1 ∪ CQ ∪ Is-lexA > Aop-GtACR1, ATR+), DL muscle calcium activity is entirely suppressed. This confirms the specificity of MN-lexA drivers and effectiveness of GtACR1 in silencing the MNs of interest.

Video S4. Only Muscle 1 remains active when MN1 is spared from silencing. In CQ ∪ Is-lexA > Aop-GtACR1, ATR+ larvae (MN1 spared), calcium activity is preserved exclusively in Muscle 1, MN1's native target. DL muscles innervated by silenced MNs remain inactive, providing functional validation of MN1's exclusive input to its native muscle-1.

Video S5. MN1-driven ectopic NMJs restore DL muscle activity following ablation. In CQ ∪ Is-lexA > Aop-RPR animals, where all native DL-innervating MNs except MN1 are ablated, GCaMP6f imaging reveals restored activity across Muscles 1, 2, 9, and 10. These data indicate that sprouted MN1 terminals form functional ectopic NMJs capable of driving muscle contractions.

Video S6. The sprouted motor axons and NMJs of bystander MN1 show GCaMP6f activity. Genotype: CQ ∪ Is-lexA > Aop-RPR; MN1-Gal4 > UAS-GCaMP6f

Video S7. Silencing the sprouted MN1 abolishes reinnervated muscle activity. In larvae expressing GtACR1 in MN1 after CQ∪Is-lexA > Aop-RPR-mediated ablation (CQ ∪ Is-lexA > Aop-RPR; MN1-Gal4 > UAS-GtACR1), light activation of GtACR1 eliminates all DL muscle activity. This confirms that reinnervation and functional output in previously denervated muscles depend on the activity of the sprouted MN1 terminals.

Video S8. Peristalsis efficiency assay comparing different genotypes. Side-by-side recordings of larval crawling behavior under different experimental conditions illustrate the impact of MN ablation, silencing, and MN1 reinnervation on locomotor efficiency. Genotypes (left to right):

1. MN1 ∪ CQ ∪ Is-lexA > Aop-GtACR1, ATR– (control)
2. MN1 ∪ CQ ∪ Is-lexA > Aop-RPR (full ablation of all DL-innervating MNs)
3. MN1 ∪ CQ ∪ Is-lexA > Aop-GtACR1, ATR+ (all DL MNs acutely silenced)
4. CQ ∪ Is-lexA > Aop-RPR (sprouted MN1 present)
5. CQ ∪ Is-lexA > Aop-GtACR1, ATR+ (MN1 intact but neighboring MNs silenced)
6. CQ ∪ Is-lexA > Aop-RPR; MN1-Gal4 > UAS-GtACR1, ATR+ (sprouted MN1 acutely silenced)

This assay visually demonstrates the functional consequences of MN ablation, silencing, and reinnervation on coordinated locomotor output.

SI References

1. Castells-Nobau, A., et al., *Two Algorithms for High-throughput and Multi-parametric Quantification of Drosophila Neuromuscular Junction Morphology.* J Vis Exp, 2017(123).
2. Nijhof, B., et al., *A New Fiji-Based Algorithm That Systematically Quantifies Nine Synaptic Parameters Provides Insights into Drosophila NMJ Morphometry.* PLoS Comput Biol, 2016. **12**(3): p. e1004823.
