# Supplementary material for: Complete muscle denervation triggers compensatory sprouting of a bystander tonic motor neuron in Drosophila larvae
Source: iScience. 2026 Jul 27;29(8):116099. doi: 10.1016/j.isci.2026.116099 (PMC13446351; doi:10.1016/j.isci.2026.116099)
Supplement: Supplementary file 2 [file mmc2.pdf]

Table S2. Staining reagents Table

Antibody	Host	Fluorophore	Dilution Factor	Source
Rabbit anti-GFP (Primary antibody)	Rabbit	n/a	1:500	Invitrogen
Mouse anti-Dlg (Primary antibody)	Mouse	n/a	1:100	DSHB
Rabbit anti mCherry/tdTomato (Primary antibody)	Rabbit	n/a	1:500	Novus
anti-F-actin (muscle)	n/a	Phalloidin CF647	1:40	Biotium
Alexa Fluor 488 Goat anti-Rabbit (Secondary antibody)	Goat	AlexaFlour488	1:200	Invitrogen
Alexa Fluor 555 Goat anti-Mouse (Secondary antibody)	Goat	AlexaFlour555	1:200	Invitrogen
Alexa Fluor 555 Goat anti-Rabbit (Secondary antibody)	Goat	AlexaFlour555	1:200	Invitrogen
Alexa Fluor 488 Goat anti-Mouse (Secondary antibody)	Goat	AlexaFlour488	1:200	Invitrogen
Alexa Fluor 405 Goat anti-Mouse (Secondary antibody)	Goat	AlexaFlour405	1:200	Invitrogen
